# Cost-effectiveness of childhood cancer treatment in Egypt: Lessons to promote high-value care in a resource-limited setting based on real-world evidence

**DOI:** 10.1016/j.eclinm.2022.101729

**Published:** 2022-11-04

**Authors:** Ranin Soliman, Jason Oke, Iman Sidhom, Nickhill Bhakta, Nancy S. Bolous, Nourhan Tarek, Sonia Ahmed, Hany Abdelrahman, Emad Moussa, Manal Zamzam, Mohamed Fawzy, Wael Zekri, Hanafy Hafez, Mohamed Sedky, Mahmoud Hammad, Hossam Elzomor, Sahar Ahmed, Madeha Awad, Sayed Abdelhameed, Enas Mohsen, Lobna Shalaby, Wael Eweida, Sherif Abouelnaga, Alaa Elhaddad, Carl Heneghan

**Affiliations:** a Department of Continuing Education, University of Oxford, UK; b Health Economics and Value Unit, Children's Cancer Hospital Egypt – 57357, Egypt; c Centre for Evidence-Based Medicine (CEBM), Nuffield Department of Primary Care Health Sciences, University of Oxford, Oxford, UK; d Paediatric Oncology Department, Children's Cancer Hospital Egypt – 57357, Egypt; e Paediatric Oncology Department, National Cancer Institute, Cairo University, Egypt; f Global Paediatric Medicine Department, St. Jude Children's Research Hospital, USA; g Clinical Oncology Department, Menoufia University, Egypt; h Paediatrics Department, National Research Centre, Cairo, Egypt; i Paediatric Oncology Department, Nasser Institute for Research and Treatment, Cairo, Egypt; j Clinical Oncology Department, Beni-suef University, Egypt; k Chief Operating Office, Children's Cancer Hospital Egypt – 57357, Egypt; l Chief Executive Office, Children's Cancer Hospital Egypt – 57357, Egypt

**Keywords:** Childhood cancer, Cost-effectiveness, Economic evaluation, DALYs, Egypt, Global health

## Abstract

**Background:**

Childhood cancer in low-and middle-income countries is a global health priority, however, the perception that treatment is unaffordable has potentially led to scarce investment in resources, contributing to inferior survival. In this study, we analysed real-world data about the cost-effectiveness of treating 8886 children with cancer at a large resource-limited paediatric oncology setting in Egypt, between 2013 and 2017, stratified by cancer type, stage/risk, and disease status.

**Methods:**

Childhood cancer costs (USD 2019) were calculated from a health-system perspective, and 5-year overall survival was used to represent clinical effectiveness. We estimated cost-effectiveness as the cost per disability-adjusted life-year (cost/DALY) averted, adjusted for utility decrement for late-effect morbidity and mortality.

**Findings:**

For all cancers combined, cost/DALY averted was $1384 (0.5 × GDP/capita), which is very cost-effective according to WHO–CHOICE thresholds. Ratio of cost/DALY averted to GDP/capita varied by cancer type/sub-type and disease severity (range: 0.1–1.6), where it was lowest for Hodgkin lymphoma, and retinoblastoma, and highest for high-risk acute leukaemia, and high-risk neuroblastoma. Treatment was cost-effective (ratio <3 × GDP/capita) for all cancer types/subtypes and risk/stage groups, except for relapsed/refractory acute leukaemia, and relapsed/progressive patients with brain tumours, hepatoblastoma, Ewing sarcoma, and neuroblastoma. Treatment cost-effectiveness was affected by the high costs and inferior survival of advanced-stage/high-risk and relapsed/progressive cancers.

**Interpretation:**

Childhood cancer treatment is cost-effective in a resource-limited setting in Egypt, except for some relapsed/progressive cancer groups. We present evidence-based recommendations and lessons to promote high-value in care delivery, with implications on practice and policy.

**Funding:**

Egypt Cancer Network; NIHR School for Primary Care Research; ALSAC.

**Research in context**

**Evidence before this study**

We considered evidence from a systematic review article of cost-effectiveness of childhood cancer treatment in LMICs published in October 2019, and we updated the evidence through April 1, 2021 by searching for additional articles, yielding 15 relevant articles. Overall, most studies reported that childhood cancer treatment was ‘cost-effective’ in LMICs, without emphasis on cancer type or risk/stage. Therefore, there is an identified gap in existing knowledge about cost-effectiveness of childhood cancer treatment using real-world data, stratified by cancer type, stage/risk, and disease status in LMIC settings.

**Added value of this study**

This study presents real-world cost-effectiveness estimates of childhood cancer treatment for 8886 children with cancer from a large paediatric oncology setting in Egypt, stratified by cancer type (ICCC-3 groups), stage/risk, and relapse/progressive disease status. We found that treatment of all childhood cancer types was either ‘very cost-effective’ or ‘cost-effective’, except for relapsed/refractory acute leukaemia which was not cost-effective based on WHO–CHOICE thresholds. We also noted that higher treatment costs were associated with inferior survival, likely due to the high costs of treating advanced-stage/high-risk and relapsed/progressive cancers, which have poor survival outcomes.

**Implications of all the available evidence**

Our data confirm prior findings that childhood cancer treatment is cost-effective in a LMIC setting, and provides new important insights with implications on practice, policy and research. Findings from our study will help clinicians make better informed decisions to provide more cost-effective treatment strategies, and will help policy-makers prioritize childhood cancer national plans. We highlight areas for future research to maximize cost-effective of treatment in priority areas, such as relapsed acute leukaemia in children.

## Introduction

Although 224,000 cases of childhood cancer were diagnosed in 2015, the global incidence was estimated to be 397,000 according to a simulation-based study.^1^ In 2017, diagnosed childhood cancers were predicted to contribute to 11.5 million disability-adjusted life years (DALY) globally based on modelled data, with 82% of this burden occurring in low- and middle-income countries (LMICs).^2^^,^^3^ Estimating DALYs from observed data is essential to determining the real-world burden of childhood cancers to better inform local policy and decision-making.^2^^,^^3^

The treatment of children with cancer is complex, resource-intensive, and incurs high costs, imposing great financial burdens on healthcare systems.^4^^,^^5^ Estimating the costs and effects/outcomes of childhood cancer treatment through cost-effectiveness analysis (CEA) will help estimate the health benefits gained from the money spent. This is especially important in LMICs because of limited resources, competing priorities, and inferior outcomes^6^^,^^7^ and will contribute to the WHO Global Initiative of Childhood Cancers (GICC) to improve survival outcomes with optimal resource use.^8^ Despite the perception that childhood cancer treatment is too costly for LMIC health systems, recent evidence shows otherwise.^3^ A systematic review by *Fung* and colleagues (2019) reported that childhood cancer treatment is very cost-effective in LMICs.^3^ Another recent study also noted that childhood cancer treatment in sub-Saharan Africa was cost-effective.^9^ Nevertheless, most of these analyses were conducted without stratifying by cancer type, risk/stage, or disease status, indicating a gap in existing literature in this area.^3^

Egypt is a lower middle-income country which has the second highest estimated number of incident childhood cancer cases in the WHO Eastern Mediterranean Region (EMR), as reported by GLOBOCAN 2020.^10^ Owing to the great need and demand for paediatric oncology services, limited resources, and relatively inferior outcomes in Egypt,^11^ there is a need to provide cost-effective childhood cancer treatment and find ways to optimise value in care delivery. Nevertheless, a gap in evidence about the costs and effects of childhood cancer treatment in Egypt exists.^3^ The Children's Cancer Hospital Egypt (CCHE) is a not-for profit paediatric oncology centre which treats around 40–50% of all childhood cancers across Egypt free-of-charge using philanthropic donations.^11^^,^^12^ Therefore, this study aims to determine the cost-effectiveness of childhood cancer treatment in a large paediatric oncology centre (CCHE) in Egypt between 2013 and 2017, by cancer type and stage/risk at diagnosis, disease status, and to determine the association between costs and survival. It also identifies the childhood cancer types/groups associated with high costs and poor survival, and provides evidence-based recommendations to promote high-value care and increase cost-effectiveness of treatment.

## Methods

### Patients and setting

We included a retrospective cohort of 8886 children with cancer (aged 0–18 years), newly diagnosed between 1st January 2013 and 31st December 2017 at the Children's Cancer Hospital Egypt (CCHE). All diagnoses were categorized according to the *International Classification of Childhood Cancer, 3rd edition* (ICCC-3)^13^ with ICD-O-3 coding (Supplementary Table S1).

**Fig. 1:**
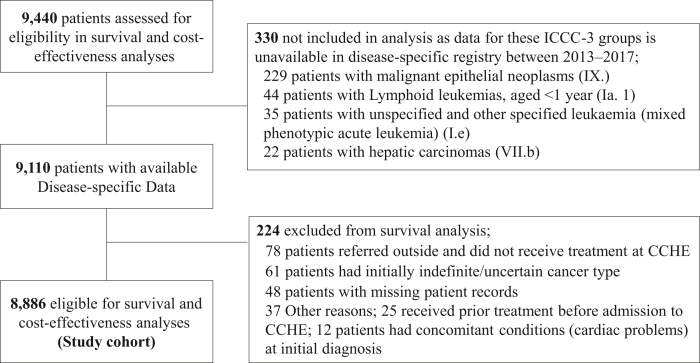
**Flowchart of included/excluded patients in survival and cost-effectiveness analyses**.

Eligible patients for cost-effectiveness/survival analysis met the following criteria: received treatment interventions at CCHE; were not lost to follow-up or referred outside CCHE early on treatment (during first 14 days from diagnosis); and had complete clinical/survival data [defined as having complete disease-related characteristics including stage/risk/subtype, updated disease status (relapse/progressive disease/refractory), and updated survival status]. Fig. 1 shows the flowchart of inclusion/exclusion criteria. The following ICCC-3 subgroups (n = 330) were excluded from survival/cost-effectiveness analyses because their clinical/survival data were incomplete and data were not readily available: (Ia.1) lymphoid leukaemia, aged <1 year; (Ie.): unspecified leukaemia; (VIIb) hepatic carcinomas and (IX) other malignant epithelial neoplasms. Eligible patients were monitored with active follow-up, and their survival data was updated until 31st December 2020. The study was approved by the scientific committee at CCHE, and the ethics approval was waived by the institutional review board because the study uses routinely-collected secondary data with deidentified patients' records.

We evaluated the cost-effectiveness of childhood cancer treatment for this cohort of patients at CCHE, because it is the largest paediatric oncology hospital in Egypt, which treats around half the children with cancer from the different areas/cities across Egypt.^12^ Besides, CCHE is the only paediatric oncology setting in Egypt with a reliable costs and survival database enabling cost-effectiveness analysis. The hospital has 320 inpatient beds and treats an average of 2000 new patients annually.^12^ CCHE provides comprehensive diagnostic and treatment services (chemotherapy, laboratory, radiology, radiotherapy, surgical oncology, neuro-surgery, orthopaedic surgery, bone marrow transplant (BMT), ICU, and palliative care).^12^ Patients are treated based on standard treatment protocols adopted from high-income countries (Supplementary Table S2, Supplementary Text S1.2), where there is waiting list of about one week for patients to be initially admitted and treated. For patients with relapsed/refractory disease, we provide second-line and/or third-line therapies based on cancer type and timing of relapse, as specified by CCHE guidelines.

### Data collection and validation

This study estimates direct medical costs from the health system perspective. Cost data were electronically captured from the readily available costing/billing database at *Oracle* system. Cost categories included personnel costs (medical and non-medical); laboratory and imaging tests; medications/drugs, surgery, radiotherapy, supplies; and overhead costs from central administrative departments and operations of inpatient/outpatient units. Definitions of cost categories and methods of measurement/allocation are described in Supplementary Table S3.

We estimated incident costs of treating children with newly diagnosed cancer at 3 years post-diagnosis, to cover costs of first-line treatment and subsequent treatment (Supplementary Text S1.3). We made cost adjustments by converting costs from the local currency (Egyptian Pounds, EGP) to US dollars (USD) using the World Bank exchange rate.^14^ We inflated to the reference year (2019) using US inflation rates based on the World Bank GDP deflator^15^ (Supplementary Text S1.3). Cost outcomes were reported in USD; whereas, the change in costs between 2013 and 2017 was conducted in local currency (and USD) due to devaluation of the EGP in 2016.^16^ Costs in EGP were adjusted to the same reference year (2019) using Egypt's inflation rates based on World Bank GDP deflator, to present changes in costs in real terms. We followed the costing methodology reported by *Soliman* and colleagues (2021), which estimated resource use/costs for the same cohort.^17^

To evaluate the health effects/outcomes of treatment, we extracted patients' demographics, disease characteristics, and survival data from CCHE hospital-based registries on the *Research Electronic Data Capture* platform.^18^ Data variables included childhood cancers by ICCC-3 groups, initial stage/risk, survival status, date of last contact, and disease status (relapse, refractory, or progressive disease [PD]). PD was defined as ‘at least a 20% growth in the size of the tumour or spread of the tumour since the beginning of treatment’, and refractory disease was defined as ‘not responding to treatment or as treatment failure’.

Cost data were validated at time of data extraction by comparing medication chart audits with the costing/billing data, with 96.5% concordance between records. Data quality checks were done every 6 months over the five years by the research department and lead paediatric oncologists at CCHE through continual monitoring and validation of the cancer registry and disease-specific registries. Disease-related and survival data were reviewed upon study initiation and validated for integrity, completeness, and accuracy. Approval for data collection was obtained from CCHE's scientific committee, and the study was exempt from institutional review board approval as it uses routinely collected secondary data and patients' records were de-identified.

### Cost-effectiveness analysis

We determined the cost-effectiveness of childhood cancer treatment compared to that of no treatment, following the assumption that children with cancer would not survive if left untreated (Supplementary Text S1.4).^1^^,^^19^^,^^20^ Cost-effectiveness was calculated by using the disability-adjusted life-year (DALY) methodology reported by *Bhakta* et al. (2013)^19^ and *Fuentes-Alabi* et al. (2018)^20^ (Supplementary Text S1.4). DALY estimates were based on years of life lost (YLL) and years lived with disability (YLD),^19^ calculated from mean age at diagnosis (for each cancer at CCHE), life expectancy in Egypt (72.06 years in 2020),^21^ and 5-year OS at CCHE. We made utility adjustments for late-effect morbidity and for excess late morbidity and mortality to avoid over-estimation of cost-effectiveness^19^ (Supplementary Text S1.4). Cost-effectiveness calculations were completed using the Excel spreadsheet for DALY Calculation Model as provided in the supplementary files from Bhakta et al. (2013).^19^

We discounted costs and future years of life saved at 3% discount rate to obtain cost per DALY (cost/DALY) averted at the base-case scenario. On sensitivity analysis, we discounted costs and effects at 6% discount rate, to allow for comparability with previously published studies, taking a similar conservative approach.^19^^,^^20^ We also conducted two-way sensitivity analyses by varying discount rates (at 0%, 3%, 6%), potential utility-adjustment for excess late-effect morbidity, and potential early mortality due to childhood cancer treatment (reduction in life expectancy at 15% and 30%)^20^ (Supplementary Text S1.5).

We used the WHO-CHOICE (CHOosing Interventions that are Cost-Effective) threshold to determine cost-effectiveness of health interventions, in terms of cost/DALY averted relative to the country's GDP per capita.^22^ The WHO threshold suggests that interventions costing <1 × the GDP per capita are ‘very cost-effective’, and those <3 × GDP per capita are ‘cost-effective’.^22^ In Egypt, GDP per capita was $3019 in 2019.^23^ We used the GDP-based DALY threshold as it is a consistent methodology allowing for fair comparisons across disease areas,^24^ and it is the most commonly used threshold to judge cost-effectiveness of interventions in LMICs lacking locally developed cost-effectiveness thresholds.^25^ This method enables comparison with findings from similar previously published studies,^3^^,^^19^^,^^20^ and provides an indication of whether, in a given setting, an intervention would represent good or poor value for the money spent (i.e. cost-effective or not).^26^

We calculated actual 5-year overall survival and cost/DALY averted for all cancers combined in the study cohort (n = 8886), and then stratified by cancer type (ICCC-3 groups), risk/stage/sub-type at diagnosis, and disease status (relapse/refractory/PD) at 3 years post–diagnosis (at a fixed interval from date of diagnosis). We did not intend to evaluate the cost-effectiveness of treatment of relapse separately, but it was evaluated as part of treatment (including first-line and second-line), with disease status stratification. Definitions of childhood cancer risk stratification and staging at CCHE are outlined (Supplementary Table S4).

The change in costs and effects of treatment over time was determined by calculating the incremental cost-effectiveness ratio (ICER), as change in cost per change in survival for patients diagnosed in 2013 and 2017 following *Lin* et al. (2016) method,^27^ (Supplementary Text S1.6). In ICER calculation, costs were estimated in EGP to account for change in the currency exchange rate and economic instability in Egypt between 2013 and 2017. We used 3-year OS because patients diagnosed in 2017 only completed 3 years of follow-up, so, 5-year OS could not be estimated.

### Statistical analysis

We analysed and reported median values and 95% CI of the cost estimates as the main descriptive statistics due to non-normality of data. We used Wilcoxon and Kruskall–Wallis tests to compare costs between the following patient groups, as appropriate: risk/stage/subtype groups, relapse/PD status, patients who underwent BMT versus those who did not, and between patients diagnosed in 2013 versus 2017. The 5-year OS (95% CI) was calculated using the Kaplan–Meier method, and we used log-rank tests to compare the difference in OS between groups. Statistical significance was determined at *p* < 0.05. Cox proportional hazards (PH) models were used to evaluate the association between treatment costs (USD) and 5-year OS, adjusting for ‘year of diagnosis’, ‘sex’, ‘age at diagnosis’, and ‘diagnostic group’ variables. We used stratified cox model using the *strata*() function for the ‘age at diagnosis’ and ‘diagnostic group’ variables, as appropriate. We also conducted the Cox PH model for each of the four main cancer groups (leukaemia, lymphoma, brain tumours, other solid tumours), adding the ‘disease status’ as a confounding factor. We tested the Cox PH model assumptions statistically and graphically using the scaled Schoenfeld residuals, and we also tested the model's goodness-of-fit using the partial log-likelihood. For Kaplan Meier and Cox regression analyses, patients were followed-up from the date of diagnosis, until either last date-of-follow-up (30th December 2020) or date of death; patients who did not complete 5 years of follow-up were censored. We derived hazard ratios (HRs), 95% confidence intervals (CIs), and coefficients from Cox models. All statistical analyses were performed in *R* statistical package version 4.0.2. by using *dplyr*, *survival*, *survminer*, and *scales* packages.

We used the *Consolidated Health Economic Evaluation Reporting Standards* (CHEERS) 2022 checklist,^28^ to outline study design and report study results (Supplementary Table S5).

### Role of the funding source

The funders had no role in the study design, data collection, data analysis, data interpretation, writing of the manuscript, or in the decision to submit the manuscript for publication. All authors had full access to the full data in the study and accept responsibility to submit for publication.

## Results

### Patient characteristics

We extracted data of 9440 children with cancer who were treated at CCHE between 2013 and 2017, of which 8886 were eligible for inclusion in the study. We analysed cost and survival data for the included 8886 patients. Table 1 shows patient and disease characteristics; the mean age at diagnosis was 6.4 years (SD ± 4.7), and male to female ratio was 1.4:1. Cancer types were distributed as follows: 26.5% had leukaemia, 15.2% had lymphomas, 18.6% had CNS/brain tumours, 37.5% had solid tumours, and 2.2% had other tumours. Three years post diagnosis, 2024 patients (22.7%) died; 120 patients (1.5%) were lost follow-up, and 1258 (14.2%) experienced relapse/refractory or PD.

**Table 1:** Patient and disease characteristics at initial diagnosis, and survival/disease status at 3 years post-diagnosis, (N=8886).

	No. of patients	Percent (%)
**Age (y) at diagnosis**		
Mean ± SD	6.4 (**±**4.7) years	
Median (IQR)	5.1 (2.5–9.7) year	
**Age-groups**		
0–1 year	732	8.2%
1–4 years	3587	40.4%
5–9 years	2444	27.5%
10–14 years	1518	17.1%
15–18 years	605	6.8%
**Gender**		
Male	5151	58%
Female	3735	42%
**Childhood cancer types, ICCC-3 groups**		
I. leukaemia	2357	26.5%
Ia. Acute lymphoblastic leukaemia	1660	18.7%
Ib. Acute myeloid leukaemia	544	6.1%
Ic. Chronic myeloid leukaemia	77	0.9%
Id. Myelodysplastic syndrome	28	0.3%
Ie. Juvenile myelomonocytic leukaemia	48	0.5%
II. Lymphomas	1344	15.1%
IIa. Hodgkin lymphoma	709	8.0%
IIb. Non-Hodgkin lymphoma	635	7.1%
III. Brain tumours	1659	18.7%
IV–X. Other solid tumours	3329	37.5%
IVa. Neuroblastoma	967	10.9%
V. Retinoblastoma	523	5.9%
VI. Renal tumours	521	5.9%
VIIa. Hepatoblastoma	122	1.4%
VIIIa. Osteosarcoma	305	3.4%
VIIIc. Ewing sarcomas	297	3.3%
IXa. Rhabdomyosarcoma	317	3.6%
IXb–d. Other soft tissue tumours	127	1.4%
Xa–c. Germ cell tumours	150	1.7%
XII. Other tumours (LCH)	197	2.2%
**Survival status**^a^		
Alive	6742	75.8%
Died	2024	22.7%
Lost follow-up (during/after treatment)^b^	120	1.5%
**Relapse/refractory or PD status**^a^		
Yes	1258	14.1%
No	7628	85.9%

Abbreviations: ICCC-3: International Classification of Childhood Cancer, 3rd edition; LCH: Langerhans cell histiocytosis. PD: Progressive Disease.

PD was defined as cancer that is growing, spreading or getting worse (at least a 20 percent growth in the size of the tumour or spread of the tumour since the beginning of treatment).

a Survival and relapse/refractory/PD status were determined at the end of 3 years post-diagnosis.

b Patients who lost follow-up are those who did not show for treatment or lost contact (after completing treatment) for at least 3 months, and were not reachable through contact by phone.

### Cost-effectiveness estimates

For all childhood cancers combined, median total 3-year costs were $19,799, and 5-year OS was 73.1% (Table 2). Cost/DALY averted for all cancers combined was $1384, which is 0.5 times the GDP per capita, thus considered “very cost-effective” per WHO–CHOICE criteria (Table 2). The highest cost/DALY averted (*i.e. least cost-effective*) was noted for patients with leukaemia, while it was lowest (*i.e. most cost-effective*) for patients with lymphoma, and retinoblastoma (range: 0.1–1.3 times GDP per capita). On sensitivity analysis at 6% discount rate, cost/DALY averted was either <1× or <3× GDP per capita. In the two-way sensitivity analyses at varying discount rates (at 0%, 3%, 6%), we found that the resultant cost/DALY averted remained below 1 × GDP per capita (i.e.: very cost-effective) (Supplementary Table S6). As our study cohort consists of a paediatric population (median age: 6 years), therefore, YLL was the major contributor in DALY calculations, whereas, YLD during treatment and late-effect morbidity had a lower impact on DALYs. The main cost categories were personnel (38.5%), medications (21.7%), and overhead costs from central administrative departments and operations of inpatient/outpatient units (25.3%) (Supplementary Table S7).

**Table 2:** Cost per DALY averted for childhood cancers (2013–2017), by ICCC-3 groups, diagnosed between 2013 and 2017 (N=8886).

Childhood cancers (No.)	5-year overall survival (%) (95% CI)	Median costs∗ (95% CI)	Cost/DALY averted (3% discount rate)∗∗	Ratio of cost/DALY averted to GDP/capita (3%)ˆ	Cost/DALY averted (6% discount)∗∗	Ratio of cost/DALY averted to GDP per capita (6%)∗∗∗
**All cancers combined (n = 8886)**	73.1 (72.1–0.74.0)	$19,799 (8921–34,204)	$1384	0.5	$2347	0.8
**I. Leukaemia (n = 2357)**	74.6 (72.7–76.4)	$35,193 (34,240–35,817)	$1762	0.6	$2904	1.0
Ia. Acute lymphoblastic leukaemia (n = 1660)	81.6 (79.7–83.5)	$33,043 (25,270–43,201)	$1512	0.5	$2505	0.8
Ib. Acute myeloid leukaemia (n = 544)	54.5 (50.2–59.2)	$43,309 (31,201–59,997)	$3033	1.0	$5077	1.7
Ic. Chronic myeloid leukaemia (n = 77)	94.8 (89.9–99.8)	$27,790 (14,149–47,499)	$1205	0.4	$1808	0.6
Id. Myelodysplastic syndrome (n = 28)	38.8 (23.8–63.3)	$45,618 (29,012–55,227)	$3898	1.3	$6521	2.2
Ie. Juvenile myelomonocytic leukaemia (n = 48)	35.3 (23.0–54.0)	$31,055 (21,404–43,632)	$4010	1.3	$7727	2.6
**II. Lymphomas (n = 1344)**	89.7 (88.0–91.4)	$10,799 (9333–12,106)	$644	0.2	$1037	0.3
IIa. Hodgkin lymphoma (n = 709)	95.2 (93.4–97.0)	$5960 (4925–7513)	$297	0.1	$464	0.2
IIb. Non-Hodgkin lymphoma (n = 635)	83.6 (80.6–86.5)	$21,509 (15,037–34,167)	$1105	0.4	$1831	0.6
**III. Brain tumours (n = 1659)**	60.8 (57.9–63.7)	$12,607 (6469–19,726)	$848	0.3	$1416	0.5
**IV–X. Other solid tumours (n=3329)**	70.4 (67.5–70.8)	$17,607 (17,156–18,494)	$1132	0.4	$2005	0.7
IVa. Neuroblastoma^ (n = 967)	55.4 (52.2–58.8)	$25,459 (14,479 38,746)	$1973	0.7	$3693	1.2
V. Retinoblastoma (n = 523)	95.1 (93.0–96.9)	$7433 (5476–9647)	$377	0.1	$725	0.2
VI. Renal tumours (n = 521)	83.1 (79.9–86.4)	$10,357 (7599–19,177)	$708	0.2	$1298	0.4
VIIa. Hepatoblastoma (n = 122)	63.4 (55.1–72.7)	$17,477 (13,106–21,760)	$1176	0.4	$2232	0.7
VIIIa. Osteosarcoma (n = 305)	46.2 (40.7–52.5)	$34,519 (22,761–42,897)	$2137	0.7	$3043	1.0
VIIIc. Ewing sarcomas (n = 297)	67.6 (62.0–73.7)	$28,398 (23,203–35,881)	$1433	0.5	$2197	0.7
IXa. Rhabdomyosarcoma (n = 317)	58.4 (52.9–64.3)	$16,607 (12,137–23,946)	$1248	0.4	$2154	0.7
IXb–d. Other soft tissue tumours (n = 127)	80.5 (73.7–87.8)	$14,829 (6646–25,538)	$721	0.2	$1157	0.4
Xa–c. Germ cell tumours (n = 150)	88.3 (83.1–93.6)	$11,984 (5136–18,348)	$560	0.2	$986	0.3
**XII.Other tumours (LCH) (n = 197)**	90.2 (86.1–94.4)	$7821 (4711–11,304)	$395	0.1	$708	0.2

∗ Median costs are estimated in US dollars.

∗∗ Costs and effects (survival) were discounted at 3% (base-case scenario), and 6% on sensitivity analysis.

∗∗∗ Ratio <1 (very cost-effective); ratio between 1 and 3 (cost-effective); Ratio >3 (not cost-effective).

^ Includes Neuroblastoma and ganglioneuroblastoma.

**Fig. 2:**
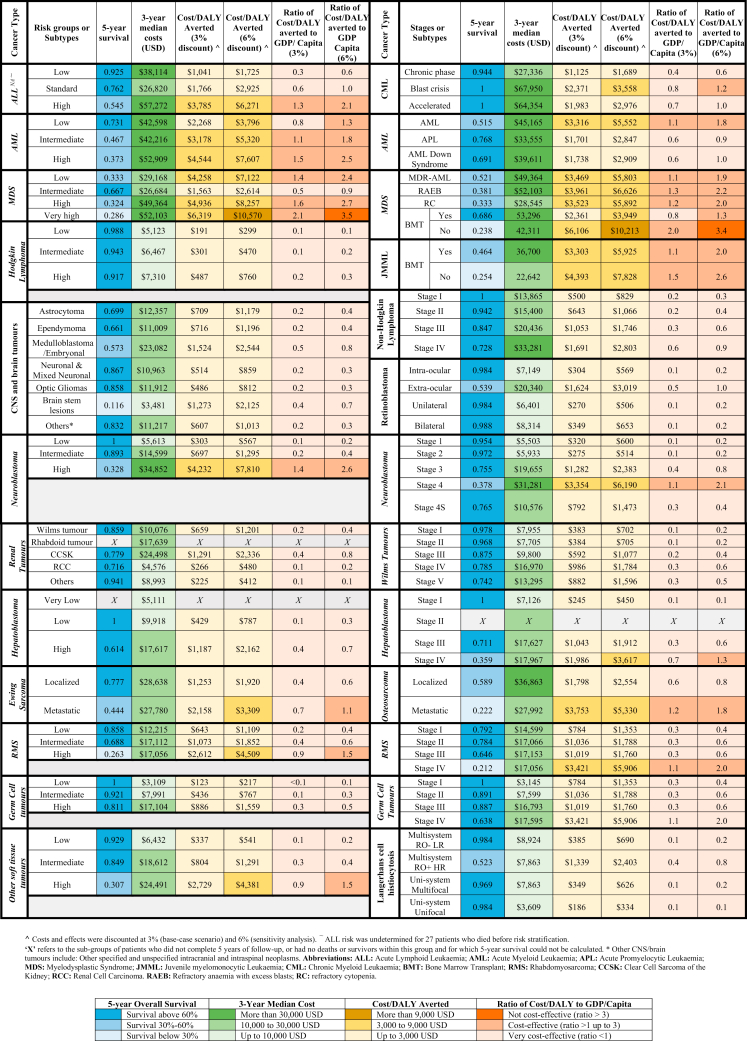
**Cost per DALY averted for childhood cancers, stratified by stage/risk, subtype, BMT status**.

**Fig. 3:**
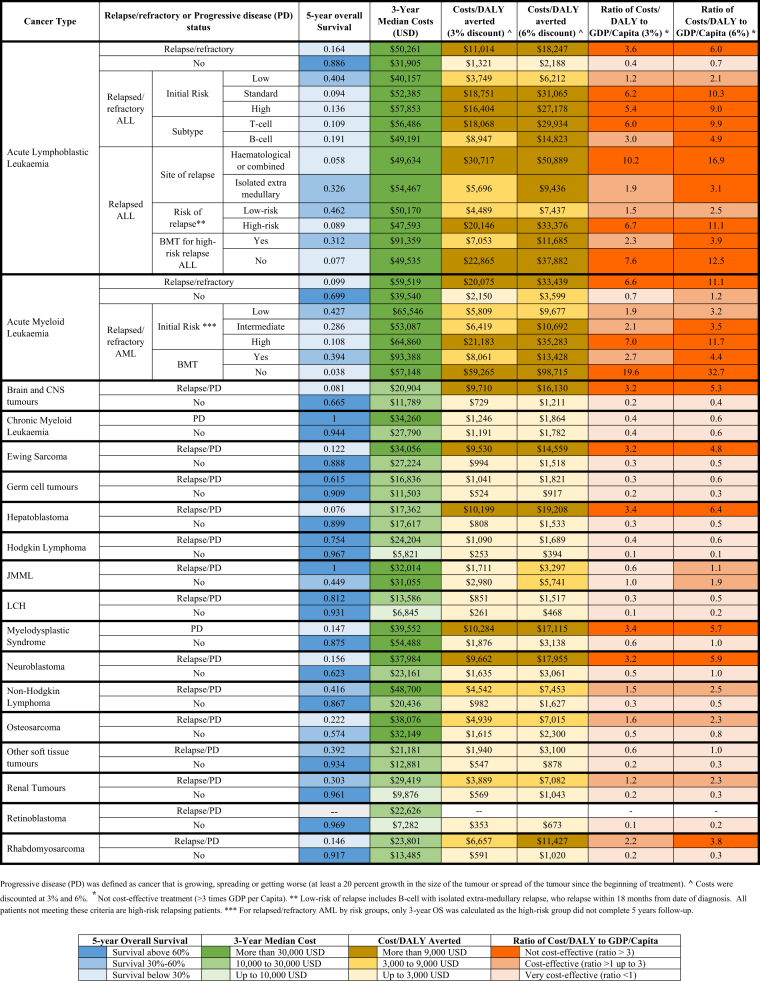
**Cost per DALY averted, stratified by relapse/refractory or progressive disease (PD) status at 3 years post-diagnosis**.

On stratifying patients by stage/risk/subtype at diagnosis, we found that cost/DALY averted varied by disease severity, with higher cost/DALY averted was noted for the high-risk and advanced stage (defined as: metastatic disease, stage IV) groups. The ratio of cost/DALY averted to GDP per capita exceeded 1 (i.e., cost-effective but not very cost-effective) for high-risk ALL, high-risk acute myeloid leukaemia (AML), advanced myelodysplastic syndrome (MDS), high-risk neuroblastoma, metastatic osteosarcoma, and metastatic rhabdomyosarcoma (Fig. 2, Supplementary Table S8). Whereas this ratio was <1 (i.e., very cost-effective) for patients with all lymphoma subtypes, intra-ocular retinoblastoma, low-risk rhabdomyosarcoma and germ cell tumour subtypes. Additionally, patients with MDS and high-risk neuroblastoma who underwent BMT had lower cost/DALY averted; more cost-effective than those who did not undergo BMT (Fig. 2, Supplementary Table S8). Patients with acute leukaemia (ALL and AML) who had relapsed/refractory disease within the first 3 years post-diagnosis (early relapse) had cost/DALY averted >3 × GDP per capita (i.e., not cost-effective) (Fig. 3, Supplementary Table S9). In relapsed/refractory acute leukaemia, cost/DALY averted was not cost-effective for patients with either ALL that was initially standard/high-risk and high-risk at time of relapse, T-cell ALL, or high-risk AML Supplementary Table S10). We found that patients with high-risk ALL at time of relapse and those with relapsed/refractory AML who underwent BMT had more cost-effective treatment than did those who did not undergo BMT (Supplementary Table S10). Furthermore, ratio of cost/DALY averted to GDP/Capita also exceeded 3 (i.e.: not cost-effective) for the patient groups with relapsed/progressive brain tumours, hepatoblastoma, Ewing sarcoma, neuroblastoma, and MDS (Fig. 3, Supplementary Table S9).

Change in cost per change in 3-year OS (ICER) is shown in Supplementary Table S11*.* Between 2013 and 2017, median costs increased by 25.0% for all cancers combined, from EGP 316,228 ($20,784) to EGP 395,302 ($20,914) (*p* < 0.001), with varying rise in costs among the cancer types. However, costs reported in USD decreased by 25.8% owing to the impact of EGP devaluation. The OS improved from 74.1% to 78.7% for all cancers combined (*p* < 0.001) and for ALL (by 6.9%, *p* = 0.007), Ewing sarcoma (by 19.5%, *p* = 0.012), non-Hodgkin lymphoma (NHL) (by 12.7%, *p* = 0.040), and hepatoblastoma by (27.4%, *p* = 0.018) (Supplementary Table S11). Yet, OS decreased for neuroblastoma by 11.3% (*p* = 0.045), and the remaining cancers showed no change in survival. The disease characteristics of the two cohorts (those diagnosed in 2013 versus 2017) are similar (Supplementary Table S12).

### Association between costs and survival

On multivariate Cox regression analysis, higher costs (each 1000 USD) were associated with inferior survival [HR (95% CI): 1.025 (1. 023–1.028); *p* < 0.001] for all cancers combined, after adjusting for potential confounders (Supplementary Table S13). Similarly, higher costs were associated significantly with worse survival outcomes within each of the four cancer groups; leukaemia, lymphoma, brain tumours, and other solid tumours**,** as follows: leukaemia [HR (95% CI): 1.012 (1.006–1.02); *p* < 0.001], lymphoma [HR (95% CI): 1.035 (1.024–1.045); *p* < 0.001], brain tumours [HR: 1.017 (1.003–1.03); *p* < 0.001], and other solid tumours [HR: 1.026 (1.023–1.03); *p* < 0.001] (Supplementary Table S13). The Cox PH assumptions were met indicating goodness-of-fit.

## Discussion

In the current study, we found that childhood cancer treatment was very cost-effective at CCHE, as the ratio of cost/DALY averted to GDP per capita (0.5) was far below the WHO–CHOICE threshold. To our knowledge, this is the first study to report the cost-effectiveness of all childhood cancer types by ICCC-3 groups in an LMIC-setting, stratified by stage/risk and relapse/progressive disease status. In aggregate, our data confirm prior findings from similar studies showing that treating childhood cancer is cost-effective in LMICs. However, by providing detailed data at the ICCC-3, risk/stage, and disease status subgroups, we provide important new insights that can help decision-makers plan therapy and prioritize treatment interventions and investments. Our study follows the recommendations reported by Fung and colleagues (2019) to conduct reliable economic evaluations in LMICs by following the CHEERS checklist in study design and reporting of results (Supplementary Table S5).^3^ Furthermore, all cost items were included, providing comprehensive treatment costs up to 3 years post-diagnosis. Another strength of the study is the availability and use of high-quality data, as data quality checks were routinely done every 6 months over the 5 years, ensuring validity of study outcomes.

Cost-effectiveness of treatment varied substantially with childhood cancer type, as the observed differences in costs vary with treatment complexity and duration, and differences in survival vary with the nature of each malignancy.^3^ Cost/DALY averted was highest for patients with AML due to intensive supportive care and long hospital stays which incur high costs and relatively inferior survival. This was also high for ALL due to longer chemotherapy duration, for osteosarcoma due to expensive orthopaedic surgeries, and for neuroblastoma due to highly intensive chemotherapy.^3^ Furthermore, acute complications of therapy and management of treatment-related toxicities also contributed to higher costs of more-intensive therapies for patients with AML and high-risk neuroblastoma. However, patients with lymphoma had low cost/DALY averted due to requiring less-intensive treatment of short-duration chemotherapy.^3^ Cost-effectiveness of treatment also varied greatly by disease severity, where the high-risk/advanced-stage cancers showed higher cost/DALY averted (Supplementary Table S8), likely attributable to their high costs of treatment and comparatively inferior survival outcomes.

Notably, treatment of relapsed/refractory acute leukaemia (ALL/AML) was not cost-effective as per the WHO-CHOICE criteria. This is likely due to high costs of treatment (first-line and second-line/third-line treatment for relapse/refractory disease) and poor survival outcomes. Furthermore, patients with indicated BMT (MDS, high-risk neuroblastoma, relapsed/refractory AML, and high-risk relapsing ALL) who underwent BMT had more cost-effective treatment than did those who were ineligible or unable to receive a transplant. Nevertheless, owing to the limited BMT bed capacity, only a small proportion of these patients who have an HLA–matched donor underwent BMT. Knowing that BMT is cost-effective has implications for future investments by CCHE to scale-up its capacity.

Between 2013 and 2017, costs of treatment (EGP) increased for all cancers combined by 25.0% in real terms after adjusting for increasing local prices due to inflation. Devaluation of EGP has increased the financial burden in Egypt by increasing the USD-based costs of imported medications, supplies, and equipment, whereas, there was no increase in personnel costs or overhead costs. The varying rise in costs for some cancers could be due to changes in treatment protocols and clinical practices. The 3-year OS improved for ALL, AML, NHL, and Ewing sarcoma due to providing more intensive supportive care, and for hepatoblastoma due to improvements in surgical techniques. Neuroblastoma OS decreased significantly, likely due to shifting to more intensive treatment for high-risk patients with more treatment-related mortalities. We do not believe that change in costs or survival over time was due to a higher percent of patients presenting with advanced disease in the later years, as the distribution of disease characteristics was similar in the two cohorts (Supplementary Table S12).

Patients with higher costs of treatment were associated with inferior survival after adjusting for confounding factors in the multivariate Cox model, likely because the high-risk/advanced-stage or relapsed/PD groups incur higher costs and have poorer survival. Although treatment is offered free-of-charge and the hospital costs are covered, families may still incur substantial non-hospital costs including costs of travel for treatment, and costs of accommodation and meals for the primary caregiver while the child is in hospital. The hospital assists parents with limited resources to cover these costs.

Our findings are in accordance with the systematic review findings by *Fung* and colleagues (2019), which showed that childhood cancer treatment was consistently very cost-effective in LMICs.^3^ In the current study, the ratio of cost/DALY averted to GDP per capita was 0.5, similar to findings by *Githang'a* and colleagues (2021) in Tanzania and Zimbabwe.^9^ The main cost categories in our study were personnel, medications, and overhead costs, which is in line with the findings of the systematic review.^3^ We found that medication costs were high as we use intensive treatment protocols adopted from high-income countries, unlike the results from Ghana, which used less-intensive protocols of lower costs.^29^

A few studies in the literature reported treatment cost-effectiveness of individual childhood cancers in LMICs, limiting comparison with our results by ICCC-3 groups. For patients with ALL, the ratio of cost/DALY averted to GDP per capita was 0.5, which is higher than that reported in other studies ranging from <0.1 to 0.25, likely due to varying protocols for different countries.^3^ In our study, this ratio was higher for Hodgkin lymphoma (0.1 versus 0.05 in South Africa), and lower for retinoblastoma (0.1 versus 0.2 in Uganda).^30^^,^^31^ There is a lack of data about cost-effectiveness of paediatric cancer treatment in the Eastern Mediterranean countries.^32^

Although abandonment of treatment is a major cause of treatment failure in LMICs, particularly where parents are required to pay out-of-pocket,^9^^,^^20^^,^^29^ this was not a problem in our centre as the abandonment rate is 1.5% and, therefore, did not likely affect our results. This low rate of abandonment is attributed to regular patients' follow-up, and patients receiving treatment free-of-charge with limited out-of-pocket expenses to the family to finance costs of travel and accommodation, as well as loss of earnings. Financing childhood cancer treatment at CCHE through philanthropy was found to be a successful funding scheme, similar to other centres in LMICs.^20^^,^^33^ Subsidizing care and making it achievable for patients across different socio-economic groups greatly contributes to improved survival outcomes, otherwise, successful treatment for childhood cancer would only become accessible to the wealthy elite.

Childhood cancer treatment at CCHE is financed by the CCHE 57357 foundation (CCHF) (https://www.57357.org/en/about-57357/overview-history/), AFNCI (https://www.afnci.org.eg/?page_id=240), and Egypt Cancer Network–USA (https://www.egyptcancernetwork.org/). Although treatment at CCHE is very cost-effective, affordability is a distinct issue as reported by *Renner* and colleagues (2018).^29^ In CCHE, the median 3-year treatment cost is $19,799 which is six times the GDP per capita, and would not have been affordable for families if they had to pay out-of-pocket, as reported in other studies.^3^^,^^29^

Despite the validity of our study methodology, some limitations exist. First, although WHO-CHOICE thresholds are informative in assessing cost-effectiveness of interventions, they are debatable as using GDP-based thresholds seems to lack country specificity. That, in addition to the uncertainty in the modelled cost-effectiveness ratios, can lead to wrong decisions about how to spend healthcare resources.^26^ Therefore, our findings should be interpreted with caution, and the WHO advised that decisions about financing should take into consideration the budget impact and affordability, in a transparent context-specific decision-making process, rather than using the threshold values separately.^26^ Other thresholds are also available for health economic evaluation, such as the marginal productivity-based threshold which provides a good option for informing decisions around healthcare resource allocation.^34^ Yet, this threshold should be country-specific to reflect national health opportunity costs^34^; nevertheless, it is lacking in most LMICs, including Egypt, limiting applicability to our context. Although some health economists consider the 3 × GDP per capita threshold too high for LMICs, treatment would still be 'cost-effective' at CCHE based on stricter thresholds.^35^ A recent review by *Kazibwe* et al. (2021) identified a stricter threshold at 0.5 × GDP per capita, which is categorized as an opportunity cost CET.^25^ Yet, the WHO-CHOICE thresholds remain the standard generic method for CEA for countries that lack local thresholds; until a more appropriate replacement is found, researchers will continue to use this GDP-based DALY threshold. Second, we included 3-year costs post-diagnosis, covering costs of all first-line treatment and early treatment failure, whereas late relapses (beyond 3 years) were not accounted for. Nevertheless, because 70% of disease relapse occurs within the first three years from diagnosis, most of relapses and their related costs are already included. We believe this will not likely affect our findings, as the ratio of cost/DALY averted to GDP/capita is far below 1, and treatment would still be cost-effective. Third, we excluded some cancer subtypes that lacked complete clinical/survival data at the time of study initiation (Fig. 1). Nevertheless, these patients represent 3.4% of the initial study cohort and excluding them would not likely impact the results. Additionally, the exclusion criteria were determined upfront before study initiation, and we did not intentionally remove any patient groups with high/low costs, good/poor survival, or advanced disease that would potentially affect outcomes. Fourth, some patients may be placed on a waiting list during treatment, affecting the dose intensity of drugs, which in turn can lead to disease relapse, impacting cost-effectiveness of treatment. Fifth, patients were followed-up until December 2020 (during the COVID-19 pandemic), so, it is possible that COVID-19 could have affected patients' outcomes due to pandemic-related restrictions; therefore, the impact of the pandemic on childhood cancer survival should be carefully considered as it may differ from one setting to another. Yet, we believe this did not greatly impact patients’ outcomes at CCHE, as there were no drug shortages or major delays in treatment during this time. Finally, this study was conducted at a single centre, so, the generalizability of our results to other centres in Egypt and other LMICs is unknown due to differences in treatment protocols and standards of care. However, to address this gap we transparently disclosed the protocols used by our centre for each cancer type.

Multiple stakeholders can leverage our findings at our centre and other centres in LMICs to inform clinical practice, policy, and future research. From a clinical perspective, determining the cost-effectiveness of treatment by ICCC-3 groups, stratified by risk/stage, and disease status would help clinicians make better informed decisions based on evidence. Additionally, this study presents novel insights into how cancer biology (type/sub-type and risk), stage, and disease status contribute to treatment cost-effectiveness, with potentially generalizable findings: patients with relapsed/progressive cancers, and advanced-stage/high risk cancers show less cost-effective treatment (despite varying costs and survival from context to another); offering BMT for the indicated patients leads to more cost-effective therapy. These interesting findings would trigger other centres in LMICs to judge the cost-effectiveness of their treatment strategies. From a policy perspective, our findings would help prioritize childhood cancer national plans and encourage policy-makers to establish locally developed cost-effectiveness thresholds, taking into consideration the budget impact and affordability. Furthermore, policy-makers in LICs can learn that investing in diagnosis and treatment facilities/infrastructure significantly improve patient outcomes in a cost-effective approach. This contributes to the WHO GICC in LMICs (Pillar 3 of the *CureAll* Framework) by optimizing regimens to deliver high-quality treatment and develop national standards of care.^8^ From a research perspective, our rigorous methodological approach creates a template that researchers in other resource-limited contexts can use in future cost-effectiveness studies. Moreover, our findings pave the way for future research to find solutions to further increase cost-effectiveness of childhood cancer treatment in priority areas.

We provide the following evidence-based recommendations to promote high-value care and increase cost-effectiveness of treatment in three perspectives: (1) clinical practice: focus on the realistic goal of improving curative outcomes for the cancers with the most cost-effective treatments (relatively low-cost and high survival outcomes); maximise cost-effectiveness of treatment by providing BMT services for the indicated patients; (2) policy-making: advocate adopting the most cost-effective treatment strategies for childhood cancers by making price negotiations and establishing collaborations with international institutions; understand the clinicians’ perception of barriers and facilitators of implementing evidence-based cost-effective interventions; (3) future research: systematically review the evidence to determine the cost-effectiveness of novel treatment strategies for relapsed acute leukaemia, other relapsed cancers, and the high-risk/advanced-stage cancers.

Childhood cancer treatment is cost-effective in a resource-limited setting in Egypt, except for relapsed/refractory acute leukaemia and other relapsed/PD cancer groups. Evaluating treatment cost-effectiveness for all childhood cancers, and stratified by stage/risk and disease status, helped us identify priority areas for improvement. Cost-effectiveness of treatment varied by disease severity, where patients with higher costs were associated with inferior survival. This is likely attributed to the high cost of treating high-risk/advanced-stage and relapsed/PD cancers, which have inferior survival. The provided evidence-based recommendations and lessons learnt will have practice, policy, and research implications to promote high-value paediatric oncology care in local context, and in other resource-limited settings in LMICs which would learn from the potentially generalizable insights obtained from our study findings.

## Contributors

Conceptualization, R.S., J.O., and C.H.; methodology, R.S., N.B., N.T., J.O., and C.H.; software, R.S.; formal analysis, R.S., N.T., J.O., and N.B.; resources, R.S. and N.S.B.; data curation, R.S. and N.T.; writing original draft preparation, R.S.; writing-review and editing, R.S., N.S.B., J.O., C.H., and A.E.; supervision, J.O., A.E., and C.H.; project administration, N.T., and N.S.B.; funding acquisition, R.S., C.H., N.B., N.S.B. Two authors (RS and NT) have verified the underlying data. All authors read and approved the final version of the manuscript.

## Data sharing statement

De-identified individual patient data for the costs and outcomes of treatment can be made available, after publication, upon request to the study PI and the corresponding author with a research proposal and signed data usage agreement. The data used and methods of data collection and analysis are described in this article. Additional study methods are in the appendix.

## Declaration of interests

RS was funded by Egypt Cancer Network (ECN) for her research work on this study. CH is funded by the National Institute for Health Research (NIHR) School for Primary Care Research [Project Number 390] and NIHR Oxford BRC. CH also received expenses for his media work and the WHO. The views expressed are those of the author(s) and not necessarily those of the NIHR, the NHS or the Department of Health. NB and NSB are funded, in part, by ALSAC. All remaining authors declared no conflicts of interest. The authors were not paid to write this article by a pharmaceutical company or other agency. The funders had no role in the study design, data collection, data analysis, data interpretation, writing of the manuscript, or in the decision to submit the manuscript for publication. All authors had full access to the full data in the study and accept responsibility to submit for publication.

## Appendix A Supplementary data

Supplementary Materials

Caption for Supplementary Materials
