# Supplementary material for: Cost-effectiveness of childhood cancer treatment in Egypt: Lessons to promote high-value care in a resource-limited setting based on real-world evidence
Source: eClinicalMedicine. 2022 Nov 4;55:101729. doi: 10.1016/j.eclinm.2022.101729 (PMC9646894; doi:10.1016/j.eclinm.2022.101729)
Supplement: Supplementary Materials [file mmc1.docx]

**Supplementary Materials**

**Cost-effectiveness of childhood cancer treatment in Egypt: Lessons to promote high-value care in resource-limited settings based on real-world evidence**

Ranin Soliman, Jason Oke, Iman Sidhom, Nickhill Bhakta, Nancy S. Bolous, Nourhan Tarek, Sonia Ahmed, Hany Abdelrahman, Emad Moussa, Manal Zamzam, Mohamed Fawzy, Wael Zekri, Hanafy Hafez, Mohamed Sedky, Mahmoud Hammad, Hossam Elzomor, Sahar Ahmed, Madeha Awad, Sayed Abdelhameed, Enas Mohsen, Lobna Shalaby, Wael Eweida, Sherif Abouelnaga, Alaa Elhaddad, Carl Heneghan

**Contents**

S1. Supplementary Methods ----------------------------------------------------------------------------- 2

Supplementary References -------------------------------------------------------------------------------- 5

Supplementary Table S1 ----------------------------------------------------------------------- ----------- 6

Supplementary Table S2 ----------------------------------------------------------------------------------- 7

Supplementary Table S3 ----------------------------------------------------------------------------------- 8

Supplementary Table S4 ----------------------------------------------------------------------------------- 9

Supplementary Table S5 ---------------------------------------------------------------------------------- 11

Supplementary Table S6 ---------------------------------------------------------------------------------- 13

Supplementary Table S7 ---------------------------------------------------------------------------------- 14

Supplementary Table S8 ---------------------------------------------------------------------------------- 15

Supplementary Table S9 ---------------------------------------------------------------------------------- 20

Supplementary Table S10 --------------------------------------------------------------------------------- 22

Supplementary Table S11 --------------------------------------------------------------------------------- 23

Supplementary Table S12 --------------------------------------------------------------------------------- 24

Supplementary Table S13 --------------------------------------------------------------------------------- 28

**S1. Supplementary Methods**

**S1.1. Coding of childhood cancer diagnoses**

Childhood cancer diagnoses were categorized based on the *International Classification of Childhood Cancer, 3rd edition* (ICCC-3), and were coded in the hospital-based cancer registry at the Children’s Cancer Hospital Egypt (CCHE) according to the WHO *International classification of diseases for oncology, third edition* (ICD-O-3) as shown in the below table.^1^

| **Childhood cancer diagnosis** | **Morphology ICD-O-3 codes** |
| --- | --- |
| Lymphoid leukaemia | 9820, 9823, 9826, 9827, 9831–9837, 9940, 9948 |
| Acute myeloid leukaemia | 9840, 9861, 9866, 9867, 9870–9874, 9891, 9895–9897, 9910, 9920, 9931 |
| Chronic myeloproliferative diseases | 9863, 9875, 9876, 9950, 9960–9964 |
| Myelodysplastic syndrome and other myeloproliferative diseases | 9945, 9946, 9975, 9980, 9982–9987, 9989 |
| Unspecified and other specified leukaemia | 9800, 9801, 9805, 9860, 9930 |
| Hodgkin lymphoma | 9591, 9670, 9671, 9673, 9675, 9678–9680, 9684, 9689–9691, 9695, 9698–9702, 9705, 9708, 9709, 9714, 9716–9719, 9727–9729, 9731–9734, 9760–9762, 9764–9769, 9970, 9687 |
| CNS and miscellaneous intracranial and intra-spinal neoplasms | 9383, 9390–9394; 9380; 9384, 9400–9411, 9420, 9421–9424, 9440–9442; 9470–9474, 9480, 9508; 9501–9504; 9380; 9381, 9382, 9430, 9444, 9450, 9451, 9460; 8270–8281, 8300, 9350–9352, 9360–9362, 9412, 9413, 9492, 9493, 9505–9507, 9530–9539, 9582; 8000–8005 |
| Neuroblastoma and ganglioneuroblastoma | 9490, 9500 |
| Retinoblastoma | 9510–9514 |
| Wilms tumour | 8959, 8960, 8964–8967 |
| Renal carcinomas | 8010–8041, 8050–8075, 8082, 8120–8122, 8130–8141, 8143, 8155, 8190–8201, 8210, 8211, 8221–8231, 8240, 8241, 8244–8246, 8260–8263, 8290, 8310, 8320, 8323, 8401, 8430, 8440, 8480–8490, 8504, 8510, 8550, 8560–8576 |
| Hepatoblastoma | 8970 |
| Osteosarcoma | 9180–9187, 9191–9195, 9200 |
| Ewing tumour and related sarcomas of bone | 9260 |
| Rhabdomyosarcoma | 8900–8905, 8910, 8912, 8920, 8991 |
| Other specified soft tissue sarcomas | 8587, 8710–8713, 8806, 8831–8833, 8836, 8840–8842, 8850–8858, 8860–8862, 8870, 8880, 8881, 8890–8898, 8921, 8982, 8990, 9040–9044, 9120–9125, 9130–9133, 9135, 9136, 9141, 9142, 9161, 9170–9175, 9231, 9251, 9252, 9373, 9581, 8830, 8963, 9180, 9210, 9220, 9240, 9260, 9364, 9365, 8800–8805 |
| Germ cell tumours | 9060–9065, 9070–9072, 9080–9085, 9100, 9101, 9060–9065, 9070–9072, 9080–9085, 9100–9105, 9060–9065, 9070–9073, 9080–9085, 9090, 9091, 9100, 9101 |

**S1.2. Adopted treatment protocols for children with cancer**

Children with cancer at our centre (CCHE) were treated based on standard treatment protocols adopted from modified international protocols from high-income countries (HICs). The majority of childhood cancer types were treated based on standard treatment protocols adopted from COG (Children’s Oncology Group).^2^ Standard treatment of children with acute lymphoblastic leukaemia (ALL) was adopted based on St. Jude Total–XV.^3^

**S1.3. Costing approach**

We estimated ‘incident costs’ of newly diagnosed children with cancer starting from the date of diagnosis, covering the first 3 years following diagnosis (3-year costs).^4^ Incident costs were chosen to cover the costs of first-line treatment and treatment failure including management of relapse/refractory or progressive disease (PD) within 3 years post-diagnosis. We made cost adjustment for inflation as per *Turner et al* methodology, which adjusts for inflation and currency changes.^5^ First, we converted costs from local currency (EGP) to US dollars (USD) using the average exchange rates for each year over the study period (between 2013 and 2017). Then we inflated using the US inflation rates to the reference year (2019).5 Also, costs in EGP were adjusted for domestic inflation using Egypt’s inflation rates to the same reference year (2019) based on World Bank GDP deflator, to present changes in costs in real terms.

**S1.4. Disability-adjusted life years (DALY) calculation**

We determined cost-effectiveness of childhood cancer treatment based on the disability-adjusted life-years (DALY) methodology used by Bhakta *et al* (2013)^6^ and Fuentes-Alabi *et al* (2018)^7^ based on the standard methods from the WHO Global Burden of Disease (GBD) working group.^8,9^ DALYs are a standard measure used to describe the number of years of life lost (YLL) due to ill health, disability or early mortality. DALY is estimated by adding the YLL with the years lived with disability (YLD).^6^ The number of YLL per case without treatment was first calculated by subtracting the standard life expectancy of Egypt (72.06 years in 2020) from the mean age at diagnosis (for each cancer at CCHE).^6^ We assumed that patients with acute leukaemia will survive for 1 month, lymphomas and other leukaemia types will survive for 3 months, and solid tumours will survive for 6 months if left untreated (in accordance with the expected clinical course of these cancers based on clinician opinion at CCHE).

We made utility adjustment for late-effect morbidity to avoid over-estimation of cost-effectiveness by obtaining the number of YLD per case through multiplying the estimated duration of disability (duration of treatment) by the disability weights for childhood cancers as set by the Global Burden of Disease (GBD) working group.^8,9^ Adjustment for late-effect morbidity was made by varying the duration of disability by cancer type, based on duration of therapy at CCHE as follows: 2.5 years for ALL, 6 months for Lymphomas, and 1 year for all other cancer types. We also adjusted duration of disability by stage for cancers that had different duration of therapy by disease stage as per the adopted protocols at CCHE. Additionally, we adjusted for excess late morbidity and mortality among childhood cancer survivors using the Medical Expenditures Panel Survey (MEPS)^12^ and Childhood Cancer Survivorship Study (CCSS)^13^ methods following the same methodology by *Nickhill et al* (2013)^6^ and *Fuentes-Alabi et al* (2018)^7^. MEPS provides utility weights generalizable to the United States general population, whereas, CCSS provides utility weights for late effects for those who received treatment for cancer in childhood.

All cost-effectiveness calculations were completed using the Excel spreadsheet for DALY Calculation Model as provided in the supplementary files from Bhakta *et al* (2013) ^6^, as a modified template of the Excel sheet.

**S1.5. Sensitivity analysis**

We discounted costs and outcomes (future years of life saved) at 3% discount rate to obtain cost per DALY (cost/DALY) averted for the base-case scenario. Sensitivity analysis was conducted by discounting costs and effects at 6%. Discounting at 6% was made for all cost per DALY calculations stratified by cancer type (ICCC-3 groups), stage/risk, and disease severity (relapse/progressive disease). Furthermore, we also conducted sensitivity analysis to adjust for potential reduction in life expectancy or late mortality due to childhood cancers.^11^ For all cancers combined, we also made sensitivity analysis by varying discount rate (0%, 3%, 6%), utility adjustment for late-effect morbidity, and reduction in life expectancy (by 15% and 30%) as a result of earlier mortality because of late-effects of treatment, following the methodology by Fuentes-Alabi et al (2018).^7^

**S1.6. ICER calculation**

Since cost-effectiveness of interventions may change over time, we determined the change in costs and effects of treatment by year of diagnosis (2013 vs. 2017). We calculated the incremental cost-effectiveness ratio (ICER) following *Lin et al* (2016) method,^14^ as incremental costs (difference between median costs of patients diagnosed in 2017 compared with those diagnosed in 2013) divided by the incremental survival (difference between the 3-year overall survival of patients diagnosed in 2017 compared with those diagnosed in 2013). ICER was estimated as change in cost per change in survival in EGP, to account for change in currency exchange rate and economic instability in Egypt between 2013–2017.

**Additional References**

1. Steliarova-Foucher E, Stiller C, Lacour B, Kaatsch P. International Classification of Childhood Cancer, third edition. *Cancer*. 2005 Apr 1;103(7):1457-67.
2. The Children’s Oncology Group (COG). Accessed on 10th July 2021 at <https://childrensoncologygroup.org/>
3. Pui CH, Relling MV, Sandlund JT, Downing JR, Campana D, Evans WE. Rationale and design of Total Therapy Study XV for newly diagnosed childhood acute lymphoblastic leukemia. *Ann Hematol*. 2004;83 Suppl 1:S124-6.
4. Barlow E. Overview of Methods to Estimate the Medical Costs of Cancer. *Med Care*. 2009 July; 47(7 Suppl 1): S33–S36.
5. Turner H, Lauer J, Tran B, Teerawattananon Y, and Jit M. Adjusting for inflation and currency changes within health economic studies. *Value Health*. 2019; 22(9):1026–1032.
6. Bhakta N, Martiniuk AL, Gupta S, Howard SC. The cost effectiveness of treating paediatric cancer in low-income and middle-income countries: a case-study approach using acute lymphocytic leukaemia in Brazil and Burkitt lymphoma in Malawi. *Arch Dis Child*. 2013;98:155-160.
7. Fuentes-Alabi S, Bhakta N, Vasquez RF, Gupta S, Horton SE. The cost and cost-effectiveness of childhood cancer treatment in El Salvador, Central America. *Cancer*. 2018 Jan 15;124(2):391-397.
8. Murray CJ, Lopez AD, World Health Organization. The global burden of disease: a comprehensive assessment of mortality and disability from diseases, injuries, and risk factors in 1990 and projected to 2020. Global Burden of Disease and Injury Series, Vol II. Cambridge, MA: Harvard School of Public Health; 1996.
9. World Health Organization. The Global Burden of Disease: 2004 Update. World Health Organization, 2008. Accessed at: <http://www.who.int/healthinfo/global_burden_disease/GBD_report_2004update_full.pdf>
10. WHO. Making Choices in Health: WHO Guide to Cost-effectiveness Analysis. WHO 2003. Geneva.
11. Armstrong GT, Yasui Y, Robison LL. Reduction in late mortality after childhood cancer. *N Engl J Med*. 2016;375:290-292.
12. Agency for Healthcare Research and Quality (AHRQ). Medical Expenditure Panel Survey. Accessed 07 May, 2022 at: <https://meps.ahrq.gov/mepsweb/>
13. Oeffinger KC, Mertens AC, Sklar CA, et al. Chronic health conditions in adult survivors of childhood cancer. *The New England journal of medicine*. 2006; 355(15):1572–1582.
14. Lin PJ, Winn AN, Parsons SK, Neumann PJ, Weiss ES, Cohen JT. Linking Costs and Survival in the Treatment of Older Adults With Chronic Myeloid Leukaemia.. *Med Care*. 2016 Apr;54(4):380-5.

**Supplementary Table S1. Childhood cancer types grouped by International Classification of Childhood Cancers, third edition (ICCC-3) diagnostic groups**

| ICCC-3 Diagnostic Groups |
| --- |
| I. Leukaemia and related cancers |
| Ia. Acute Lymphoid Leukaemia |
| Ib. Acute Myeloid Leukaemia |
| Ic. Chronic Myeloid Leukaemia |
| Id. (subset) Myelodysplastic Syndrome |
| Id. (subset) Juvenile myelomonocytic Leukaemia |
| Ie. Unspecified and other specified leukemia |
| II. Lymphomas |
| IIa. Hodgkin Lymphoma |
| IIb. Non-Hodgkin Lymphoma |
| IIIa-e CNS tumours |
| IIIa. Ependymomas and choroid plexus tumours |
| IIIb. Astrocytoma |
| IIIc.1-2 Medulloblastoma/Embryonal CNS tumours |
| IV.a Neuroblastoma and ganglioneuroblastoma |
| V. Retinoblastoma |
| VI. Renal tumours |
| VIa.1 Wilms tumour |
| VIa.2 Rhabdoid renal tumours |
| VIa.3 Kidney sarcomas |
| VII. Hepatic tumours |
| VII.a Hepatoblastoma |
| VII.b Hepatic carcinomas |
| VIII. Malignant bone tumours |
| VIIIa Osteosarcoma |
| VIIIc Ewing sarcomas |
| IX. Soft tissue and extra osseous sarcoma |
| IXa. Rhabdomyosarcoma |
| IXb-d Other soft tissue tumours |
| X. a-c Germ Cell tumours |
| XI. Other malignant epithelial neoplasms and malignant melanomas |
| XII. Other and unspecified malignant neoplasms* includes LCH* |

* XII includes LCH only. Abbreviations. LCH. Langerhans cell histiocytosis.

**Supplementary Table S2. Standard Treatment protocols adopted at CCHE**

| ICCC-3 Diagnostic Groups | Standard treatment adopted from these protocols |
| --- | --- |
| I. Leukaemia and related cancers | |
| Ia. Acute Lymphoid Leukaemia | St. Jude Total XV protocol (1^st^ line treatment); St. Jude ALL R16 protocol (for relapsed/refractory disease) |
| Ib. Acute Myeloid Leukaemia | COG [AAML0531; COG AAML1031; COG AAML1831]  Relapsed/refractory AML: FLAG-M |
| Ic. Chronic Myeloid Leukaemia | European Leukaemia Net (ELN) guidelines |
| Id. Myelodysplastic Syndrome | Bone Marrow Transplant (BMT) |
| Id. Juvenile myelomonocytic Leukaemia | COG [2x FLA cycle followed by Azacitidine 6 cycles;  Maintenance: LDARA-C, 6MP; 13-cis-Retinoic acid; BMT] |
| II. Lymphomas | |
| IIa. Hodgkin Lymphoma | ABVD Regimen |
| IIb. Non-Hodgkin Lymphoma | COG ANHL0131 (ALCL); LMB-96 (Mature B NHL); ALL total XV (LBL) |
| IIIa-e CNS tumours | |
| IIIa. Ependymomas and choroid plexus tumours | CCG-9942 |
| IIIb. Astrocytoma | COG A9952 (LGG); CCG-943 (HGG); COG ACNS0126 (HGG) |
| IIIc.1-2 Medulloblastoma/Embryonal CNS tumours | COG-ACNS0331 (SR-MB); ACNS0332 (HR-MB); P9934 (Inf-MB); DFCI 02-294 (ATRT) |
| IV.a Neuroblastoma | COG A3973 (HR); SFOP VP16-Carbo/CADO (IR) |
| V. Retinoblastoma | COG ARET0332 (HR); COGARET0231 (Groups C and D); COGARET0331 (Group B); COG ARET0321 (extra-ocular) |
| VI. Renal tumours | |
| VIa.1 Wilms tumour | COG AREN0532 and COG AREN0533 (unilateral); COG AREN0534 (bilateral) |
| VIa.2 Rhabdoid renal tumours | COG AREN0321 |
| VIa.3 Kidney sarcomas | COG AREN0321 (clear cell sarcoma of kidney) |
| VII. Hepatic tumours |  |
| VII.a Hepatoblastoma | COG AHEP0731 |
| VIII. Malignant bone tumours | |
| VIIIa Osteosarcoma | EURAMOS-1 (ISRCTN67613327) |
| VIIIc Ewing sarcomas | POG #9354/CCG #7942; COG AEWS0031; COG |
| IX. Soft tissue and extra osseous sarcoma | |
| IXa. Rhabdomyosarcoma | COG ARST0531; COG ARST0331 (LR); COG-D9802 |
| IXb-d Other soft tissue tumours | COG & NRG ONCOLOGY ARST1321 |
| X. a-c Germ Cell tumours | AGCT01P1 (HR) / AGCT0132 (LR/SR) |
| XI. Other malignant epithelial neoplasms and malignant melanomas | |
| XII. Langerhans cell histiocytosis | LCH IV protocol from the Histiocyte Society |
| Management of Fever neutropenia | NCCN and IDSA guidelines |

**Abbreviations:** **COG:** Children’s Oncology Group; **ELN:** European Leukaemia Net; **LBL:** Lymphoblastic Lymphoma; **ALCL:** Anaplastic large cell lymphoma; **CCG:** Children’s Cancer Group; **LGG:** Low-grade Glioma; **HGG:** High-grade Glioma; **MB:** Medulloblastoma: **ATRT:** Atypical Teratoid Rhabdoid tumour; **HR:** High-Risk; **SR:** Standard Risk; **LR:** Low Risk; **IR:** Intermediate Risk; **POG:** Paediatric Oncology Group; **NCCN:** National Comprehensive Cancer Network; **IDSA** Infectious Disease Society of America.

**Supplementary Table S3. Definitions of cost categories/sub-categories, and methods of measurement and/or allocation**

| Cost categories | Definition | How it is measured/allocated? |
| --- | --- | --- |
| Personnel costs   - Medical ^a^ - Non-medical ^b^ | Include costs (salaries) of all medical personnel directly related to patient care, and non-medical personnel providing administrative and managerial functions.  Full salaries are included in personnel costs, as all medical and non-medical personnel are 100% full-time dedicated to paediatric oncology care at CCHE. | Personnel costs are allocated to patients by dividing total personnel costs for each unit by these allocation keys (per month): number of patients (inpatient units); number of visits (outpatient units); number of surgeries (surgery unit); number of radiotherapy sessions (radiotherapy unit); number of lab tests (lab unit); number of imaging tests (imaging unit). |
| Investigation costs:  Laboratory costs | Costs of all laboratory and pathology tests including costs of materials (kits, reagents, others), facilities (electricity, water, gas), and maintenance/depreciation of equipment in the lab unit. | Cost data of all laboratory/pathology tests per patient, as captured from the costing database/Oracle. |
| Investigation costs:  Imaging costs | Costs of all imaging tests including costs of materials (contrast, FDG, anaesthesia), facilities (mentioned above), and maintenance/depreciation of equipment in the radiology/nuclear medicine units. | Cost data of all imaging tests per patient, as captured from the costing database/Oracle. |
| Treatment interventions cost: Medications/drugs costs | Costs of all medications/drugs including both chemotherapy and supportive care drugs. **^^^** | Cost data of all medications/drugs, per patient, as captured from the costing database/Oracle. |
| Treatment cost:  Surgery costs | Costs of surgery incurred in the operating room (OR) including materials, drugs/anaesthesia, facilities (mentioned above), and maintenance/depreciation of equipment in the surgery unit. | Cost data of surgery and/or radiotherapy, per patient as captured from the costing database/Oracle. Costs of drugs, materials, anaesthesia are directly allocated to the patient, and other costs are estimated for the whole department/unit, then a proportion is allocated to the patient based on:   - Type of surgery (major/minor) - Number of radiotherapy sessions/month |
| Treatment cost:  Radiotherapy costs | Costs of radiotherapy sessions including materials, drugs/anaesthesia, facilities (mentioned above), and maintenance/depreciation of equipment in the unit. |  |
| Treatment cost:  Medical supplies costs | Costs of medical supplies consumed by the patient during treatment in the inpatient or outpatient units. | Costs of supplies directly consumed by patient, as captured from costing database/Oracle. |
| Overhead costs | Costs that are not directly related to patient care and costs of operations of the inpatient and outpatient departments (that are not incurred in any of above-mentioned cost categories):   1. Total costs of central administrative departments **^c^** 2. Operational costs in the inpatient and outpatient units including: facilities (electricity, water, gas), space cost, and maintenance/depreciation costs for the inpatient and outpatient departments. [Treatment costs in the inpatient/outpatient units are included under the various treatment categories headings]. | 1. Total costs of central administrative departments are allocated to inpatient and outpatient units, based on flat rate (distributed equally among each unit), then allocated to the patient based on number of patients (inpatient units) and number of visits (outpatient units). 2. Total overhead costs (assigned to inpatient and outpatient units from administrative departments and those estimated from operations of inpatient/outpatient) are then allocated to the patient based on number of patients (inpatient units) and the number of visits (outpatient units). |

**^a^** Medical personnel costs include: paediatric oncologists, radiation oncologists, clinical pharmacists, nurses, oncology surgeons, neuro-surgeons, orthopaedic surgeons, physicians in multi-specialty outpatient clinics, intensivists, anaesthesiologists, pathologists, laboratory and imaging consultants and technicians. **^b^** Non-medical personnel include departmental secretaries, data entry specialists, and administrative managers for the inpatient, outpatient, pharmacy, laboratory, pathology, imaging, surgery, and radiotherapy departments. **^c^** Includes total costs (salaries, non-medical materials, facilities) for the central administrative departments which serve the whole hospital and are not directly related to patient care, including finance, Information Technology (IT), Human Resources (HR), purchasing, biomedical engineering, quality, research, catering, security, housekeeping, laundry, learning & development departments.

**Supplementary Table S4. Definitions of stage at diagnosis, risk stratification and sub-type classifications with references.**

| Diagnostic Group | Risk/stage classification | Definition | Reference for definitions |
| --- | --- | --- | --- |
| Ia. Acute Lymphoid Leukemia | Risk | Pui, et al. 2009. | 1. Pui, C. et al. Treating childhood acute lymphoblastic leukemia without cranial irradiation. [N Engl J Med.](https://www.ncbi.nlm.nih.gov/pubmed/19553647) 2009 Jun 25;360(26):2730-41. |
| Ib. Acute Myeloid Leukaemia | Subtype | Creutzig, et al. 2012 | 1. Creutzig, U. et al. Diagnosis and management of acute myeloid leukemia in children and adolescents: recommendations from an international expert panel. Blood. 2012 18 October 120;16. |
|  | Risk | COG. AAML1031. 2018. | 1. COG. AAML1031: A Phase III Randomized Trial for Patients with de novo AML using Bortezomib and Sorafenib for Patients with High Allelic Ratio FLT3/ITD. Spring 2018 Progress Report. |
| Id. Myelodysplastic Syndrome | Risk | IPSS. 2012. | 1. Greenberg, P., et al. Revised International Prognostic Scoring System for Myelodysplastic Syndromes. Blood 2012 120:2454-2465. |
|  | Subtype | ACS. 2018. | 1. American Cancer Society. Types of Myelodysplastic Syndromes. ACS. Last Revised: January 22, 2018. Retrieved from: <https://www.cancer.org/cancer/myelodysplastic-syndrome/about/mds-types.html> |
| Ic. Chronic Myeloid leukemia | Subtype | ASCO. 2018. | 1. American Society of Clinical Oncology (ASCO). Leukemia - Chronic Myeloid - CML: Phases. Last Revised: March, 2018. Retrieved from: <https://www.cancer.net/cancer-types/leukemia-chronic-myeloid-cml/phases> |
| IIa. Hodgkin Lymphoma | Risk | Ferrari, et al. 2008 | 1. Re A, Ferrari S, and Frata P, et al. Late Computed Tomography Scan Response Improvement and Gallium Scintigraphy Evaluation as On-Treatment Prognostic Parameters to Tailor Treatment Intensity in Patients with Hodgkin's Lymphoma. *Ann Oncol*, 2008. 19(5), 951-7 |
| IIb. Non-Hodgkin Lymphoma | Staging | Modified Murphy staging | 1. Murphy SB. Classification, staging and end results of treatment of childhood non-Hodgkin's lymphomas: dissimilarities from lymphomas in adults. Semin Oncol. 1980;7:332-339. |
| IIIa-e CNS Tumours | Subtype | WHO. 2016 | 1. Louis, D. et al. The 2016 World Health Organization Classification of Tumors of the Central Nervous System: a summary. Acta Neuropathol. 2016. |
| IV.a Neuroblastoma | Risk | Davidoff. 2012 | 1. Davidoff AM. Neuroblastoma. Semin Pediatr Surg. 2012;21(1):2–14. doi:10.1053/j.sempedsurg.2011.10.009. |
|  | Staging | INSS | American cancer society. International Neuroblastoma Staging System (INSS). Last Revised: March 19, 2018. Retrieved from: <https://www.cancer.org/cancer/neuroblastoma/detection-diagnosis-staging/staging.html#references> |
| V. Retinoblastoma | Staging | IRSS | 1. Chantada, F. Doz, C.B. Antoneli, et al., A proposal for an international retinoblastoma staging system, Pediatr. Blood Cancer 47 (2006) 801–805. |
| VI. Renal Tumours | Subtype | ICCC-3 | 1. Steliarova-Foucher E, Stiller C, Lacour B, Kaatsch P. International Classification of Childhood Cancer, third edition. Cancer. 2005 Apr 1;103(7):1457-67. |
| VIa.1 Wilms Tumour | Staging | Metzger, et al. 2005 | 1. M.L. Metzger, J.S. Dome, Current therapy for Wilms’ tumor, Oncologist 10 (2005) 815–826. |
| VII.a Hepatoblastoma | Staging | Meyers, 2017 | 1. Meyers RL, Maibach R, Hiyama E, et al. Risk-stratified staging in paediatric hepatoblastoma: a unified analysis from the Children's Hepatic tumors International Collaboration. *Lancet Oncol*. 2017;18(1):122–131. |
|  | Risk | McCarville, 2012 | 1. McCarville, M. Diagnosis and Staging of Hepatoblastoma: Imaging Aspects. Pediatr Blood Cancer 2012;59:793–799. |
| VIIIc Ewing sarcomas | Staging | ASCO, 2018 | 1. American Society of Clinical Oncology (ASCO). Ewing Sarcoma - Childhood and Adolescence: Stages Approved on January 2018. Retrieved from: <https://www.cancer.net/cancer-types/ewing-sarcoma-childhood-and-adolescence/stages> |
| VIIIa Osteosarcoma | Staging | ASCO, 2018 | 1. American Society of Clinical Oncology (ASCO). Osteosarcoma - Childhood and Adolescence: Stages. Approved on January 2018. Retrieved from: <https://www.cancer.net/cancer-types/osteosarcoma-childhood-and-adolescence/stages> |
| IXa. Rhabdomyosarcoma | Risk | COG. 2012 | 1. Malempati S, Hawkins DS. Rhabdomyosarcoma: review of the Children's Oncology Group (COG) Soft-Tissue Sarcoma Committee experience and rationale for current COG studies. Pediatr Blood Cancer. 2012;59(1):5–10. |
|  | Staging | ASCO, 2018 | 1. ASCO. Rhabdomyosarcoma - Childhood: Stages and Groups. Approved on October 2018. Retrieved from: <https://www.cancer.net/cancer-types/rhabdomyosarcoma-childhood/stages-and-groups> |
| IXb-d Other soft tissue tumours | Risk | COG. 2015 | 1. Waxweiler, T., et al. Non-Rhabdomyosarcoma Soft Tissue Sarcomas in Children: A Surveillance, Epidemiology, and End Results Analysis Validating COG Risk Stratifications. [Int J Radiat Oncol Biol Phys.](https://www.ncbi.nlm.nih.gov/pubmed/25968827) 2015 Jun 1;92(2):339-48. |
| X. a-c Germ Cell Tumours | Staging | ASCO. 2018 | 1. ASCO. Germ Cell Tumor - Childhood: Stages. Approved on January 2018. Retrieved from: <https://www.cancer.net/cancer-types/germ-cell-tumor-childhood/stages> |
|  | Risk | Meisel, et al, 2015.  Gilligan, 2019 | 1. Meisel, J., et al. [Development of a risk stratification system to guide treatment for female germ cell tumors](https://ezproxy-prd.bodleian.ox.ac.uk:2073/science/article/pii/S0090825815300524). Gynecologic Oncology 138 (2015) 566–572. 2. [Gilligan, T.](https://www.uptodate.com/contents/initial-risk-stratified-treatment-for-advanced-testicular-germ-cell-tumors/contributors) and Kantoff, P. Initial risk-stratified treatment for advanced testicular germ cell tumors. Up-To-Date 2019. Retrieved from: <https://www.uptodate.com/contents/initial-risk-stratified-treatment-for-advanced-testicular-germ-cell-tumors> |
| XII. Other Tumours (LCH)* | Risk | Krooks, et al. 2018 | 1. Krooks, J. et al. Langerhans cell histiocytosis in children. Journal of American academy of dermatology. 78:6. 2018. |

* XII includes LCH only. Abbreviations. LCH. Langerhans cell histiocytosis.

**Supplementary Table S5. The Consolidated Health Economic Evaluation Reporting Standards (CHEERS) 2022 checklist**

| **Section/item** | **Item No.** | **Section/item** | **Reported**  **on page no./line no.** |
| --- | --- | --- | --- |
| **Title and abstract** | | | |
| Title | 1 | Identify the study as an economic evaluation and specify the interventions being compared. | Page 1; line 1 |
| Abstract | 2 | Provide a structured summary that highlights context, key methods, results, and alternative analyses. | Page 2 |
| **Introduction** |  |  |  |
| Background and  objectives | 3 | Give the context for the study, the study question, and its practical relevance for decision making in policy or practice. | Page 5,6; lines 103-130; Page 6 lines 130-136 |
| **Methods** | | | |
| Health economic analysis plan | 4 | Indicate whether a health economic analysis plan was developed and where available. | NA |
| Study population | 5 | Describe characteristics of the study population (such as age range, demographics, socioeconomic, or clinical characteristics). | Page 6; lines 139-147 |
| Setting and location | 6 | Provide relevant contextual information that may influence findings. | Page 7, lines 155-167 |
| Comparators | 7 | Describe the interventions or strategies being compared and why chosen. | Page 9; lines 203 - 205 |
| Perspective | 8 | State the perspective(s) adopted by the study and why chosen. | Page 7, line 169 |
| Time horizon | 9 | State the time horizon for the study and why appropriate. | Page 6; lines 139,140 |
| Discount rate | 10 | Report the discount rate(s) and reason chosen. | Page 9, 10; lines 214-220 |
| Selection of outcomes | 11 | Describe what outcomes were used as the measure(s) of benefit(s) and harm(s). | Page 9; lines 203-209 |
| Measurement of outcomes | 12 | Describe how outcomes used to capture benefit(s) and harm(s) were measured. | Page 9; lines 209-213 |
| Valuation of outcomes | 13 | Describe the population and methods used to measure and value outcomes. | Page 9,10; lines 203 - 238 |
| Measurement and valuation of resources and costs | 14 | Describe how costs were valued. | Page 7, 8; lines 169 - 185 |
| Currency, price date, and conversion | 15 | Report the dates of the estimated resource quantities and unit costs, plus the currency and year of conversion. | Page 8, lines 175 - 183 |
| Rationale and description of model | 16 | If modelling is used, describe in detail and why used. Report if the model is publicly available and where it can be accessed. | NA |
| Analytics and assumptions | 17 | Describe any methods for analysing or statistically transforming data, any extrapolation methods, and approaches for validating any model used. | Page 9; lines 203, 204 |
| Characterising heterogeneity | 18 | Describe any methods used for estimating how the results of the study vary for subgroups. | Page 10; lines 232 - 238 |
| Characterising distributional effects | 19 | Describe how impacts are distributed across different individuals or adjustments made to reflect priority populations. | NA |
| Characterising uncertainty | 20 | Describe methods to characterise any sources of uncertainty in the analysis. | Page 9, 10; lines 215 - 220 |
| Approach to engagement with patients and others affected by the study | 21 | Describe any approaches to engage patients or service recipients, the general public, communities, or stakeholders (such as clinicians or payers) in the design of the study. | NA |
| **Results** | | | |
| Study parameters | 22 | Report all analytic inputs (such as values, ranges, references) including uncertainty or distributional assumptions. | Page 12; lines 275 – 282; Page 13; lines 284 - 297 |
| Summary of main results | 23 | Report the mean values for the main categories of costs and outcomes of interest and summarise them in the most appropriate overall measure. | Page 12 – 15 |
| Effect of uncertainty | 24 | Describe how uncertainty about analytic judgments, inputs, or projections affect findings. Report the effect of choice of discount rate and time horizon, if applicable. | Page 13; lines 288 – 295 |
| Effect of engagement with patients and others affected by the study | 25 | Report on any difference patient/service recipient, general public, community, or stakeholder involvement made to the approach or findings of the study | NA |
| **Discussion** | | | |
| Study findings, limitations, generalisability, and current knowledge | 26 | Report key findings, limitations, ethical or equity considerations not captured, and how these could affect patients, policy, or practice. | Pages 15 – 22 |
| **Other** | | | |
| Source of funding | 27 | Describe how the study was funded and any role of the funder in the identification, design, conduct, and reporting of the analysis | Page 23; lines 509 – 513 |
| Conflicts of interest | 28 | Report authors conflicts of interest according to journal or International Committee of Medical Journal Editors requirements. | Page 23; lines 523, 524 |

**Supplementary Table S6. Base-case and sensitivity analysis for Cost per DALY averted, (N=8,886)**

| **Scenarios of Life Expectancy (LE)* and Late Effect Morbidity** | **Cost/ DALY averted**  **(Ratio of cost/DALY averted to GDP/capita)** | | |
| --- | --- | --- | --- |
|  | **0% discount** | **3% discount** | **6% discount** |
| Optimal Case (Normal LE, No Utility Adjustment for Late Effect Morbidity) | $478 (0.1) | $1,245 (0.4) | $2,289 (0.7) |
| Base Case (Normal LE + Utility Adjustment for Late Effect Morbidity) | $535 (0.1) | $1,384 (0.4) | $2,347 (0.7) |
| 15% Reduction in LE + Utility Adjustment for Late Effect Morbidity | $683 (0.2) | $1,507 (0.5) | $2,482 (0.8) |
| 30% Reduction in LE + Utility Adjustment for Late Effect Morbidity | $819 (0.2) | $1,662 (0.5) | $2,579 (0.8) |

*Decrements in Life Expectancy selected based on Armstrong *et al*.

**Supplementary Table S7. Childhood cancer costs at 3-years post-diagnosis (2013–2017), by major cost categories (N=8,886)**

| **Cost category** | **Median total cost (USD) per patient** | **Total cost for all patients combined** | **Percentage from total cost (%)** |
| --- | --- | --- | --- |
| Medical personnel | $7,071 | $74,297,559 | 28.8% |
| Non-medical personnel | $2,382 | $25,030,728 | 9.7% |
| Total Personnel costs | 9,453 | $99,328,287 | 38.5% |
| Laboratory costs | $1,517 | $19,652,820 | 7.6% |
| Imaging costs | $243 | $2,652,683 | 1.0% |
| Investigation costs* | $489 | $22,305,503 | 8.6% |
| Medication costs | $2,851 | $56,029,560 | 21.7% |
| Surgery/radiotherapy costs | $224 | $1,917,206 | 0.7% |
| Medical supplies costs | $915 | $13,281,560 | 5.2% |
| Treatment interventions costs** | $942 | $71,228,326 | 27.6% |
| Overhead costs | $5,736 | $65,004,711 | 25.3% |
| Sum of total costs | $19,799 | $257,866,827 | 100% |

***** Investigation costs includes laboratory and imaging costs. ****** Treatment interventions costs include costs of medications, surgery/radiotherapy, medical supplies.

**Supplementary Table S8. Cost per DALY averted for childhood cancers, stratified by stage, risk, or subtype (N=8,886)**

| **Cancer Type** | **Stages, risk, subtypes groups** | **No. of patients (%)** | **3-year costs (USD)** | | **5-year overall survival (%)** | | **Cost/DALY averted (3% discount) ^** | **Ratio of Cost/DALY averted to GDP/Capita (3%)** | **Cost/DALY averted (6% discount) ^** | **Ratio of cost/DALY averted to GDP per Capita (6%)** |
| --- | --- | --- | --- | --- | --- | --- | --- | --- | --- | --- |
|  |  |  | **Median Costs**  **(95% CI)** | ***p*-value^~^** | **Survival (95% CI)** | ***p*-value°** |  |  |  |  |
| **Acute Lymphoid Leukaemia, *n=1,660*** | | | |  |  |  |  |  |  |  |
| *Risk ^a^* | Low risk | 720 | $26,820 (51,023 – 62,825) | <0.001* | 92.5 (90.5 - 94.6) | <0.001* | $1,041 | 0.3 | $1,725 | 0.6 |
|  | Standard risk | 815 | $38,114 (37,256 – 38,977) |  | 76.2 (73.3 - 79.3) |  | $1,766 | 0.6 | $2,925 | 1.0 |
|  | High risk | 125 | $57,272 (51,023 – 62,825) |  | 54.5 (45.8 - 65.0) |  | $3,785 | 1.3 | $6,271 | 2.1 |
| **Acute Myeloid Leukaemia, *n= 544*** | | | |  |  |  |  |  |  |  |
| *Sub-type ^b^* | AML | 477 | $45,165 (42,592 – 47,507) | <0.001* | 51.5 (46.8 - 56.7) | 0.02 | $3,316 | 1.1 | $5,552 | 1.8 |
|  | APL | 38 | $33,555 (26,491 – 39,637) |  | 76.8 (66.5 - 92.9) |  | $1,701 | 0.6 | $2,847 | 0.9 |
|  | AML Down Syndrome | 29 | $39,611 (28,478 – 45,613) |  | 69.1 (54.0 - 88.0) |  | $1,738 | 0.6 | $2,909 | 1.0 |
| *Risk ^c NA ~^* | Low | 165 | $42,598 (41,186 – 46,686) | 0.003* | 73.1 (66.1 - 80.7) | <0.001* | $2,268 | 0.8 | $3,796 | 1.3 |
|  | Intermediate | 263 | $42,216 (37,963 – 45,165) |  | 46.7 (40.2 - 54.0) |  | $3,178 | 1.1 | $5,320 | 1.8 |
|  | High | 89 | $52,909 (44,710 – 59,662) |  | 37.3 (28.3 - 49.1) |  | $4,544 | 1.5 | $7,607 | 2.5 |
| **Myelodysplastic Syndrome, *n= 28*** | | | | |  |  |  |  |  |  |
| *Risk  ^d^* | Low | 3 | 29,168 (*NA*) | 0.1 | 33.3 (6.7 – 100) | 0.71 | $4,258 | 1.4 | $7,122 | 2.4 |
|  | Intermediate | 6 | $26,684 (7,722 – 71,669) |  | 66.7 (37.9 – 100) |  | $1,563 | 0.5 | $2,614 | 0.9 |
|  | High | 12 | $49,364 (32,974 – 56,116) |  | 32.4 (13.1 - 80.4) |  | $4,936 | 1.6 | $8,257 | 2.7 |
|  | Very high | 7 | $52,103 (39,552 – 73,137) |  | 28.6 (8.9 - 92.2) |  | $6,319 | 2.1 | $10,570** | 3.5** |
| *Sub-type ^e^* | MDR-AML | 6 | $49,364 (44,783 – 56,116) | 0.002* | 50.0 (22.5 – 100) | 0.66 | $3,469 | 1.1 | $5,803 | 1.9 |
|  | RAEB | 15 | $52,103 (25,487 – 56,116) |  | 38.1 (19.6 - 74.2) |  | $3,961 | 1.3 | $6,626 | 2.2 |
|  | RC | 7 | $28,545 (7,722 – 82,245) |  | 33.3 (10.8 – 100) |  | $3,523 | 1.2 | $5,892 | 2.0 |

| Did BMT? | Yes | 10 | $53,296 (25,487 – 70,071) | 0.01* | 68.6 (44.5 – 100) | 0.049* | $2,361 | 0.8 | $3,949 | 1.3 |  |
| --- | --- | --- | --- | --- | --- | --- | --- | --- | --- | --- | --- |
|  | No | 18 | $42,311 (28,545 – 53,356) |  | 23.8 (10.1 - 55.9) |  | $6,106 | 2.0 | $10,213** | 3.4** |  |
| **Juvenile myelomonocytic Leukaemia, n= 48** | | | |  |  |  |  |  |  |  |  |
| *Did BMT?* | Yes | 25 | $36,700 (29,472 – 47,516) | <0.001* | 46.4 (31.7 - 75.7) | 0.072 | $3,303 | 1.1 | $5,925 | 2.0 |  |
|  | No | 23 | $22,642 (16,705 – 32,883) |  | 25.4 (9.7 - 49.2) |  | $4,393 | 1.5 | $7,828 | 2.6 |  |
| **Chronic Myeloid Leukaemia, *n= 77*** | | | |  |  |  |  |  |  |  |  |
| *Sub-type ^f^* | Chronic phase | 72 | $27,336 (20,972 – 34,198) | 0.01* | 94.4 (89.3 - 99.9) | 0.87 | $1,125 | 0.4 | $1,689 | 0.6 |  |
|  | Blast crisis | 4 | $67,950 (*NA*) |  | 100 (100) |  | $2,371 | 0.8 | $3,558 | 1.2 |  |
|  | Accelerated | 1 | $64,354 (*NA*) |  | 100 (100) |  | $1,983 | 0.7 | $2,976 | 1.0 |  |
| **Hodgkin Lymphoma, *n=* 7*09*** | | | |  |  |  |  |  |  |  |  |
| *Risk ^g^* | Low | 348 | $5,123 (4,981 – 5,314) | <0.001* | 98.8 (97.7 - 99.9) | <0.001* | $191 | 0.1 | $299 | 0.1 |  |
|  | Intermediate | 153 | $6,467 (6,210 – 6,726) |  | 94.3 (90.1 - 98.7) |  | $301 | 0.1 | $470 | 0.2 |  |
|  | High | 208 | $7,310 (6,715 – 7,913) |  | 90.0 (85.6 - 94.7) |  | $487 | 0.2 | $760 | 0.3 |  |
| **Non-Hodgkin Lymphoma, (*n= 635)*** | | | |  |  |  |  |  |  |  |  |
| *Stage ^h^* | Stage I | 24 | $13,865 (5,928 – 18,286) | <0.001* | 100 (100) | <0.001* | $500 | 0.2 | $829 | 0.3 |  |
|  | Stage II | 87 | $15,400 (13,257 – 16,888) |  | 94.2 (89.4 - 99.3) |  | $643 | 0.2 | $1,066 | 0.4 |  |
|  | Stage III | 364 | $20,436 (19,215 – 22,070) |  | 84.7 (80.9 - 88.7) |  | $1,053 | 0.3 | $1,746 | 0.6 |  |
|  | Stage IV | 160 | $33,281 (30,104 – 35,809) |  | 72.8 (66.2 - 80.1) |  | $1,691 | 0.6 | $2,803 | 0.9 |  |
| **CNS tumours, *n= 1,659*** | | | | | | | | | | | |
| *Subtype ^I^* | Astrocytoma | 482 | $12,357 (11,370–13,309) | <0.001* | 69.9 (61.2 – 75) | <0.001* | $709 | 0.2 | $1,179 | 0.4 |  |
|  | Ependymoma | 149 | $11,009 (10,155–13,516) |  | 66.1 (55.9 - 78.1) |  | $716 | 0.2 | $1,196 | 0.4 |  |
|  | Medulloblastoma/Embryonal | 359 | $23,082 (21,447 – 24,598) |  | 57.3 (51.4 - 63.9) |  | $1,524 | 0.5 | $2,544 | 0.8 |  |
|  | Neuronal & Mixed Neuronal | 97 | $10,963 (11,370–13,309) |  | 86.7 (79.1 – 95.0) |  | $514 | 0.2 | $859 | 0.3 |  |
|  | Optic Gliomas | 92 | $11,912 (8,465 – 12,434) |  | 85.0 (76.2 - 94.9) |  | $486 | 0.2 | $812 | 0.3 |  |
|  | Brain stem lesions | 239 | $3,481 (3,178 – 3,765) |  | 11.6 (7.6 - 17.8) |  | $1,273 | 0.4 | $2,125 | 0.7 |  |
|  | Others*** | 241 | 11,217 (3,178 – 3,765) |  | 80.0 (73.3 - 87.4) |  | $607 | 0.2 | $1,013 | 0.3 |  |
| ***Neuroblastoma (NBL), n= 967*** | | | |  |  |  |  |  |  |  |  |
| *Risk  ^j^* | Low | 72 | 5,613 (4,353 – 6,338) | <0.001* | 96.9 (91.2 – 100) | <0.001* | $303 | 0.1 | $567 | 0.2 |  |
|  | Intermediate | 296 | 14,599 (13,567 – 15,822) |  | 89.3 (85.7 - 92.9) |  | $697 | 0.2 | $1,295 | 0.4 |  |
|  | High | 599 | 34,852 (32,704 – 35,897) |  | 32.8 (28.9 - 37.2) |  | $4,232 | 1.4 | $7,810 | 2.6 |  |
| BMT for HR NBL? | Yes | 140 | 45,924 (32,515 – 43,827) | <0.001 | 64.4 (18.7 - 27.4) | <0.001* | $2,785 | 0.9 | $5,139 | 1.7 |  |
|  | No | 459 | 31,119 (23,725 – 27,094) |  | 22.6 (56.2 - 73.8) |  | $5,595 | 1.9 | $10,325** | 3.4** |  |
| *Stage ^k^* | Stage 1 | 35 | 5,503 (3,928 – 6,284) | <0.001 | 95.0 (85.9 – 100) | <0.001* | $320 | 0.1 | $600 | 0.2 |  |
|  | Stage 2 | 38 | 5,933 (4,311 – 7,489) |  | 97.2 (92 – 100) |  | $275 | 0.1 | $514 | 0.2 |  |
|  | Stage 3 | 286 | 19,655 (17,773 – 22,305) |  | 75.5 (70.6 - 80.7) |  | $1,282 | 0.4 | $2,383 | 0.8 |  |
|  | Stage 4 | 565 | 31,281 (29,829 – 32,515) |  | 37.8 (33.7 - 42.4) |  | $3,354 | 1.1 | $6,190 | 2.1 |  |
|  | Stage 4S | 43 | 10,576 (7,417 – 15,200) |  | 76.0 (62.6 - 92.2) |  | $792 | 0.3 | $1,473 | 0.4 |  |
| **Retinoblastoma, *n= 523*** | | | |  |  |  |  |  |  |  |  |
| *Stage ^l^* | Intra-ocular | 479 | 7,149 (6,774 – 7,448) | <0.001* | 98.4 (97.1 - 99.6) | <0.001* | $304 | 0.1 | $569 | 0.2 |  |
|  | Extra-ocular | 44 | 20,340 (18,027 – 23,457) |  | 53.9 (40.2 - 72.2) |  | $1,624 | 0.5 | $3,019 | 1.0 |  |
| *Intra-ocular* | Unilateral | 275 | 6,401 (6,115 – 6,785) | <0.001* | 98.0 (96.3 - 99.8) | 0.44 | $270 | 0.1 | $506 | 0.2 |  |
|  | Bilateral | 204 | 8,314 (7,797 – 8,664) |  | 98.8 (97.2 – 100) |  | $349 | 0.1 | $653 | 0.2 |  |
| **Renal tumours, *n= 521*** | |  |  |  |  |  |  |  |  |  |  |
| *Sub-type* | Wilms tumours | 445 | 10,076 (9,718 – 10,567) | <0.001* | 85.9 (82.6 - 89.2) | <0.001* | $659 | 0.2 | $1,201 | 0.4 |  |
|  | Rhabdoid tumour | 13 | 17,639 (9,542 – 27,637) |  | *X* |  | *X* | *X* | *X* | *X* |  |
|  | Clear Cell Sarcoma Kidney | 35 | 24,498 (19,616 – 29,901) |  | 77.9 (64.4 - 94.3) |  | $1,291 | 0.4 | $2,336 | 0.8 |  |
|  | Renal Cell Carcinoma | 11 | 4,576 (3,155 – 9,346) |  | 71.6 (48.8 – 100) |  | $266 | 0.1 | $480 | 0.2 |  |
|  | Mesoblastic nephroma | 17 | 8,993 (3,186 – 13,066) |  | 94.1 (83.6 – 100) |  | $225 | 0.1 | $412 | 0.1 |  |
| ***Wilms t*umours, n=445** | |  |  |  |  |  |  |  |  |  |  |
| *Stage ^n^* | Stage I | 45 | 7,955 (6,603 – 9,718) | <0.001* | 97.8 (93.6 – 100) | <0.001* | $383 | 0.1 | $702 | 0.2 |  |
|  | Stage II | 68 | 7,705 (6,957 – 8,504) |  | 96.8 (96.8 - 92.6) |  | $384 | 0.1 | $705 | 0.2 |  |
|  | Stage III | 202 | 9,800 (9,157 – 10,388) |  | 87.5 (82.8 - 92.4) |  | $592 | 0.2 | $1,077 | 0.4 |  |
|  | Stage IV | 87 | 16,970 (14,278 – 20,037) |  | 78.5 (69.8 - 88.1) |  | $986 | 0.3 | $1,784 | 0.6 |  |
|  | Stage V | 43 | 13,295 (10,357 – 22,094) |  | 74.2 (62.1 - 88.6) |  | $882 | 0.3 | $1,596 | 0.5 |  |
| **Hepatoblastoma, *n= 122*** | | | |  |  |  |  |  |  |  |  |
| *Stage* *^o^* | Stage I | 4 | 7,126 (NA) | 0.02* | 100 (100) | 0.001* | $245 | 0.1 | $450 | 0.1 |  |
|  | Stage II | 1 | 15,145 (NA) |  | *X* |  | *X* | *X* | *X* | *X* |  |
|  | Stage III | 87 | 17,627 (16,537 – 19,536) |  | 71.1 (61.9 - 81.6) |  | $1,043 | 0.3 | $1,912 | 0.6 |  |
|  | Stage IV | 30 | 17,967 (13,036 – 20,480) |  | 35.9 (22.1 - 58.3) |  | $1,986 | 0.7 | $3,617 | 1.3 |  |
| *Risk ^p^* | Very Low | 1 | 5,111 (NA) | 0.03* | *X* | 0.24 | *X* | *X* | *X* | *X* |  |
|  | Low | 5 | 9,918 (NA) |  | 100 (100) |  | $429 | 0.1 | $787 | 0.3 |  |
|  | High | 116 | 17,617 (16,537 – 19,421) |  | 61.4 (52.9 - 71.3) |  | $1,187 | 0.4 | $2,162 | 0.7 |  |
| **Ewing Sarcoma, *n= 297*** | | | |  |  |  |  |  |  |  |  |
| *Stage ^q^* | Localized | 211 | 28,638 (27,288 – 30,116) | 0.4 | 77.7 (71.9 - 83.9) | <0.001* | $1,253 | 0.4 | $1,920 | 0.6 |  |
|  | Metastatic | 86 | 27,780 (24,678 – 30,445) |  | 44.4 (34.3 - 57.4) |  | $2,158 | 0.7 | $3,309 | 1.1 |  |
| **Osteosarcoma, (*n=* 305)** | | | |  |  |  |  |  |  |  |  |
| *Stage ^r^* | Localized | 199 | 36,863 (34,372 – 38,162) | 0.001* | 58.9 (52.1 - 66.4) | <0.001* | $1,798 | 0.6 | $2,554 | 0.8 |  |
|  | Metastatic | 106 | 27,992 (21,177 – 33,378) |  | 22.2 (15.2 - 32.4) |  | $3,753 | 1.2 | $5,330 | 1.8 |  |
| **Rhabdomyosarcoma, *n= 317*** | | | |  |  |  |  |  |  |  |  |
| *Risk ^s^* | Low | 25 | 12,215 (10,568 – 17,325) | 0.08 | 85.8 (75.7 - 97.2) | <0.001* | $643 | 0.2 | $1,109 | 0.4 |  |
|  | Intermediate | 214 | 17,112 (15,992 – 18,767) |  | 68.8 (63 - 74.9) |  | $1,073 | 0.4 | $1,852 | 0.6 |  |
|  | High | 78 | 17,056 (14,948 – 18,923) |  | 26.3 (18.4 - 37.6) |  | $2,612 | 0.9 | $4,509 | 1.5 |  |
| *Stage ^t^* | Stage I | 62 | 14,599 (12,607 – 18,068) | 0.3 | 79.2 (69.3 - 90.5) | <0.001* | $784 | 0.3 | $1,353 | 0.4 |  |
|  | Stage II | 27 | 17,066 (13,084 – 24,993) |  | 78.4 (62.6 - 98.1) |  | $1,036 | 0.3 | $1,788 | 0.6 |  |
|  | Stage III | 150 | 17,153 (15,826 – 19,028) |  | 64.6 (57.2 - 73.0) |  | $1,019 | 0.3 | $1,760 | 0.6 |  |
|  | Stage IV | 78 | 17,056 (14,576 – 19,150) |  | 21.2 (13.1 - 34.5) |  | $3,421 | 1.1 | $5,906 | 2.0 |  |
| **Other soft tissue tumours, *n= 127*** | | | |  |  |  |  |  |  |  |  |
| *Risk ^u^* | Low | 45 | 6,432 (4,547 – 9,966) | <0.001* | 92.9 (85.6 – 100) | <0.001* | $337 | 0.1 | $541 | 0.2 |  |
|  | Intermediate | 63 | 18,612 (15,450 – 23,108) |  | 84.9 (76.4 - 94.5) |  | $804 | 0.3 | $1,291 | 0.4 |  |
|  | High | 19 | 24,491 (14,172 – 34,751) |  | 30.7 (15.4 - 61.1) |  | $2,729 | 0.9 | $4,381 | 1.5 |  |
| **Germ Cell tumours, *n=* *150*** | | | |  |  |  |  |  |  |  |  |
| *Stage ^v^* | Stage I | 44 | 3,145 (2,698 – 3,803) | <0.001* | 100 (100) | <0.001* | $141 | <0.1 | $248 | 0.1 |  |
|  | Stage II | 20 | 7,599 (6,586 – 10,424) |  | 89.1 (75.8 – 100) |  | $403 | 0.1 | $709 | 0.2 |  |
|  | Stage III | 63 | 16,793 (14,394 – 18,900) |  | 88.7 (81.2 - 96.9) |  | $759 | 0.3 | $1,335 | 0.4 |  |
|  | Stage IV | 23 | 17,595 (14,424 – 26,008) |  | 63.8 (46.5 - 87.4) |  | $1,256 | 0.4 | $2,211 | 0.7 |  |
| *Risk ^w,x^* | Low | 42 | 3,109 (2,671 – 3,670) | <0.001* | 100 (100) | 0.008* | $123 | <0.1 | $217 | 0.1 |  |
|  | Intermediate | 27 | 7,991 (6,586 – 11,402) |  | 92.1 (82.2 – 100) |  | $436 | 0.1 | $767 | 0.3 |  |
|  | High | 81 | 17,104 (14,733 – 19,084) |  | 81.1 (72. 9 - 90.2) |  | $886 | 0.3 | $1,559 | 0.5 |  |
| **Langerhans cell histiocytosis, n= 197** | | | |  |  |  |  |  |  |  |  |
| *Risk ^y^* | Multisystem RO- LR | 51 | 8,924 (8,172 – 10,955) | <0.001* | 98.0 (94.31 – 100) | <0.001* | $385 | 0.1 | $690 | 0.2 |  |
|  | Multisystem RO+ HR | 32 | 7,863 (10,180 – 18,827) |  | 52.3 (37.4 - 73.1) |  | $1,339 | 0.4 | $2,403 | 0.8 |  |
|  | Uni-system Multifocal | 50 | 7,863 (6,233 – 9,334) |  | 96.0 (90.7 – 100) |  | $349 | 0.1 | $626 | 0.2 |  |
|  | Uni-system Unifocal | 64 | 3,609 (3,609 – 5,124) |  | 98.4 (95.3 – 100) |  | $186 | 0.1 | $334 | 0.1 |  |

**^** Costs and effects (survival) were discounted at 3% (base-case scenario), and 6% on sensitivity analysis. ** Not cost-effective treatment (>3 GDP per Capita). **^~^** P-value for difference in costs was obtained from Wilcoxon and Kruskal Wallis tests as appropriate. **°** *P-*value for difference in survival was obtained from log-rank test. ** Statistically significant at p<0.005. *** Other CNS/brain tumors include: Other specified and unspecified intracranial and intraspinal neoplasms. ***^NA^*** refers to ‘not available’, defined as patients with undetermined risk at diagnosis, as these patients died before risk determination, as follows: AML risk (n=27, 4.9%). **‘X’** refers to the sub-groups of patients who did not complete 5 years of follow-up, or had no deaths or survivors within this group, and for which 5-year survival could not be calculated. **Abbreviations: APL:** Acute Promyelocytic Leukaemia; **BMT:** Bone Marrow Transplant; **RAEB:** Refractory anaemia with excess blasts; **RC:** refractory cytopenia. ^a–y^ refer to references for definitions of staging, risk stratification and sub-type classifications systems are listed in **Supplementary Table S4**.

**Supplementary Table S9. Cost per DALY averted for childhood cancers, stratified by relapse/refractory (R/R) or progressive disease (PD) status**

| **Cancer Type** | **No. of patients (n)** | **Relapse/refractory or PD status** | **3-Year Median Costs (95% CI)** | ***p*-value^~^** | **5-year overall survival (95% CI)** | ***p*-value°** | **Costs/DALY averted (3% discount)** | **Ratio of Costs/ DALY to GDP per Capita (3%)** | **Costs/DALY averted (6% discount)** | **Ratio of Costs/DALY to GDP per Capita (6%)** |
| --- | --- | --- | --- | --- | --- | --- | --- | --- | --- | --- |
| Acute Lymphoblastic Leukaemia | 166 | R/R | 50,261 (46,882 – 53,450) | <0.001 | 0.164 (0.111 – 0.249) | <0.001 | $11,014* | 3.6* | $18,247* | 6.0* |
|  | 1,494 | No | 31,905 (31,248 – 32,700) |  | 0.886 (0.869 – 0.903) |  | $1,321 | 0.4 | $2,188 | 0.7 |
| Acute Myeloid Leukaemia | 163 | R/R | 59,519 (55,931 – 64,860) | <0.001 | 0.099 (0.049 – 0.201) | <0.001 | $20,075* | 6.6* | $33,439* | 11.1* |
|  | 381 | No | 39,540 (37,631 – 41,603) |  | 0.699 (0.654 – 0.747) |  | $2,150 | 0.7 | $3,599 | 1.2 |
| Brain and CNS tumours | 146 | Relapse/PD | 20,904 (18,490 – 22,982) | <0.001 | 0.081 (0.039 – 0.637) | <0.001 | $9,710* | 3.2* | $16,130* | 5.3* |
|  | 1,513 | No | 11,789 (11,163 – 12,490) |  | 0.665 (0.637 – 0.695) |  | $729 | 0.2 | $1,211 | 0.4 |
| Chronic Myeloid Leukaemia | 6 | PD | 34,260 (22,341 – 77,353) | 0.3 | 1 (1-1) | 0.45 | $1,246 | 0.4 | $1,864 | 0.6 |
|  | 71 | No | 27,790 (20,145 – 38,351) |  | 0.944 (0.892 – 0.999) |  | $1,191 | 0.4 | $1,782 | 0.6 |
| Ewing Sarcoma | 84 | Relapse/PD | 34,056 (30,973 – 37,526) | <0.001 | 0.122 (0.060 – 0.250) | <0.001 | $9,530* | 3.2* | $14,559* | 4.8* |
|  | 213 | No | 27,224 (26,025 – 28,392) |  | 0.888 (0.842 – 0.937) |  | $994 | 0.3 | $1,518 | 0.5 |
| Germ cell tumours | 13 | Relapse/PD | 16,836 (5,389 – 34,459) | 0.3 | 0.615 (0.400 – 0.946) | <0.001 | $1,041 | 0.3 | $1,821 | 0.6 |
|  | 137 | No | 11,503 (8,702 – 13,482) |  | 0.909 (0.860 – 0.959) |  | $524 | 0.2 | $917 | 0.3 |
| Hepatoblastoma | 42 | Relapse/PD | 17,362 (13,036 – 21,324) | 0.9 | 0.076 (0.021 – 0.271) | <0.001 | $10,199* | 3.4* | $19,208* | 6.4* |
|  | 80 | No | 17,617 (15,859 – 19,451) |  | 0.899 (0.835 – 0.968) |  | $808 | 0.3 | $1,533 | 0.5 |
| Hodgkin Lymphoma | 46 | Relapse/PD | 24,204 (16,149 – 31,052) | <0.001 | 0.754 (0.620 – 0.918) | <0.001 | $1,090 | 0.4 | $1,689 | 0.6 |
|  | 663 | No | 5,821 (5,617 – 5,977) |  | 0.967 (0.952 – 0.982) |  | $253 | 0.1 | $394 | 0.1 |
| JMML | 10 | Relapse/PD | 32,014 (25,997 – 52,340) | 0.2 | 1 (0.016 – 0.642) | 0.074 | $1,711 | 0.6 | $3,297 | 1.1 |
|  | 38 | No | 31,055 (21,720 – 36,971) |  | 0.449 (0.312 – 0.647) |  | $2,980 | 1.0 | $5,741 | 1.9 |
| LCH | 49 | Relapse/PD | 13,586 (11,968 – 16,374) | <0.001 | 0.812 (0.708 – 0.931) | 0.021 | $851 | 0.3 | $1,517 | 0.5 |
|  | 148 | No | 6,845 (5,728 – 7,542) |  | 0.931 (0.891 – 0.973) |  | $261 | 0.1 | $468 | 0.2 |
| Myelodysplastic Syndrome | 19 | PD | 39,552 (28,545 – 53,356) | 0.3 | 0.140 (0.041 – 0.480) | <0.001 | $10,284* | 3.4* | $17,115* | 5.7* |
|  | 9 | No | 54,488 (27,882 – 58,060) |  | 0.875 (0.673 – 1) |  | $1,876 | 0.6 | $3,138 | 1.0 |
| Neuroblastoma | 143 | Relapse/PD | 37,984 (34,180 – 40,425) | <0.001 | 0.156 (0.101 – 0.243) | <0.001 | $9,662* | 3.2* | $17,955* | 5.9* |
|  | 824 | No | 23,161 (21,730 – 24,457) |  | 0.623 (0.590 – 0.659) |  | $1,635 | 0.5 | $3,061 | 1.0 |
| Non-Hodgkin Lymphoma | 43 | Relapse/PD | 48,700 (39,570 – 63,171) | <0.001 | 0.416 (0.291 – 0.594) | <0.001 | $4,542 | 1.5 | $7,453 | 2.5 |
|  | 592 | No | 20,436 (19,215 – 21,792) |  | 0.867 (0.839 – 0.896) |  | $982 | 0.3 | $1,627 | 0.5 |
| Osteosarcoma | 104 | Relapse/PD | 38,076 (34,680 – 40,579) | <0.001 | 0.222 (0.149 – 0.333) | <0.001 | $4,939 | 1.6 | $7,015 | 2.3 |
|  | 201 | No | 32,149 (28,633 – 34,981) |  | 0.574 (0.507 – 0.649) |  | $1,615 | 0.5 | $2,300 | 0.8 |
| Other soft tissue tumours | 35 | Relapse/PD | 21,181 (14,172 – 26,602) | 0.001 | 0.39 (0.248 – 0.614) | <0.001 | $1,940 | 0.6 | $3,100 | 1.0 |
|  | 92 | No | 12,881 (9,966 – 17,365) |  | 0.934 (0.898 – 0.993) |  | $547 | 0.2 | $878 | 0.3 |
| Renal Tumours | 58 | Relapse/PD | 29,419 (25,054 – 35,279) | <0.001 | 0.303 (0.201 – 0.457) | <0.001 | $3,889 | 1.2 | $7,082 | 2.3 |
|  | 463 | No | 9,876 (9,354 – 10,277) |  | 0.901 (0.873 – 0.928) |  | $569 | 0.2 | $1,043 | 0.3 |
| Retinoblastoma | 10 | Relapse/PD | 22,626 (15,960 – 39,964) | <0.001 | -- | <0.001 | -- | - |  | - |
|  | 513 | No | 7,282 (7,047 – 7,711) |  | 0.969 |  | $353 | 0.1 | $673 | 0.2 |
| Rhabdomyosarcoma | 137 | Relapse/PD | 23,801 (22,148 – 26,711) | <0.001 | 0.146 (0.091 – 0.233) | <0.001 | $6,657 | 2.2 | $11,427* | 3.8* |
|  | 180 | No | 13,485 (12,553 – 15,088) |  | 0.910 (0.869 – 0.953) |  | $591 | 0.2 | $1,020 | 0.3 |

Progressive disease (PD) was defined as cancer that is growing, spreading or getting worse (at least a 20 percent growth in the size of the tumour or spread of the tumour since the beginning of treatment). * Not cost-effective treatment (> 3 times GDP per Capita, $3,019).

**^~^** P-value for difference in costs was obtained from Wilcoxon and Kruskall Wallis as appropriate.

**°** *P-*value for difference in survival was obtained from log-rank test. * Statistically significant at p<0.005.

**Supplementary Table S10. Cost per DALY averted for relapsed acute leukaemia (ALL/AML), by disease characteristics and BMT status**

| **Cancer Type** | | **No. of patients**  **(n)** | **3-Year Median Costs**  **(95% CI)** | ***p*-value^~^** | **5-year Overall Survival (95% CI)** | ***p*-value°** | **Costs/DALY averted (3% discount)** | **Ratio of Cost/DALY to GDP per Capita (3%)** | **Costs/DALY averted (6% discount)** | **Ratio of Cost/DALY to GDP per Capita (6%)** |
| --- | --- | --- | --- | --- | --- | --- | --- | --- | --- | --- |
| **Relapsed/refractory ALL (n=166)** | | | | | | | | | | |
| **Initial Risk** | Low | 35 | 40,157 (29,135 – 50,353) | <0.001 | 0.404 (0.245 – 0.629) | <0.001 | $3,749 | 1.2 | $6,212 | 2.1 |
|  | Standard | 97 | 52,385 (46,808 – 56,771) |  | 0.094 (0.045 – 0.198) |  | $18,751* | 6.2* | $31,065* | 10.3* |
|  | High | 34 | 57,853 (48,091 – 66,280) |  | 0.136 (0.0514 – 0.361) |  | $16,404* | 5.4* | $27,178* | 9.0* |
| **Subtype** | T-cell | 48 | 56,486 (46,882 – 67,069) | 0.006 | 0.109 (0.046 – 0.294) | <0.001 | $18,068* | 6.0* | $29,934* | 9.9* |
|  | B-cell | 118 | 49,191 (44,905 – 52,981) |  | 0.191 (0.121 – 0.295) |  | $8,947 | 3.0 | $14,823* | 4.9* |
| **Relapsed ALL (n=163)** | | | | | | | | | | |
| **Site of relapse** | Haematological or combined | 101 | 49,634 (45,528 – 52,983) | 0.1 | 0.058 (0.021 – 0.160) | <0.001 | $30,717* | 10.2* | $50,889* | 16.9* |
|  | Isolated extra medullary | 62 | 54,467 (46,763 – 59,527) |  | 0.326 (0.216 – 0.493) |  | $5,696 | 1.9 | $9,436* | 3.1 |
| **Risk of relapse**** | Low-risk | 27 | 50,170 (46,882 – 54,163) | 0.5 | 0.462 (0.291 – 0.732) | <0.001 | $4,489 | 1.5 | $7,437 | 2.5 |
|  | High-risk | 136 | 47,593 (37,103 – 60,355) |  | 0.089 (0.047 – 0.171) |  | $20,146* | 6.7* | $33,376* | 11.1* |
| **BMT for high-risk relapse ALL** | Yes | 10 | 91,359 (44,522 – 111,583) | <0.001 | 0.312 (0.102 – 0.955) | 0.054 | $7,053 | 2.3 | $11,685* | 3.9* |
|  | No | 126 | 49,535 (46,563 – 52,983) |  | 0.077 (0.038 – 0.160) |  | $22,865* | 7.6* | $37,882* | 12.5* |
| **Relapsed/refractory AML (n=163)** | | | | | | | | | | |
| **Initial Risk ***** | Low | 25 | 65,546 (57,449 – 82,856) | <0.001 | 0.42 (0.252 – 0.693) | 0.003 | $5,809 | 1.9 | $9,677 | 3.2 |
|  | Intermediate | 97 | 53,087 (45,665 – 59,875) |  | 0.26 (0.187 – 0.383) |  | $6,419 | 2.1 | $10,692 | **3.5*** |
|  | High | 41 | 64,860 (57,939 – 72,736) |  | 0.11 (0.04 – 0.354) |  | $21,183* | 7.0* | $35,283* | 11.7* |
| **BMT** | Yes | 25 | 92,511 (72,674 – 101,347) | <0.001 | 0.39 (0.218 – 0.727) | <0.001 | $8,061 | 2.7 | $13,428* | 4.4* |
|  | No | 138 | 55,250 (47,706 – 58,623) |  | 0.03 (0.005 – 0.162) |  | $59,265* | 19.6* | $98,715* | 32.7* |

***** Not cost-effective treatment (> 3 times GDP per Capita, $3,019). **^~^** *P*-value for difference in costs was obtained from Wilcoxon and Kruskall Wallis as appropriate. **°** *P-*value for difference in survival was obtained from log-rank test. * Statistically significant at p<0.005. ** Low-risk of relapse includes B-cell with isolated extra-medullary relapse, who relapse within 18 months from date of diagnosis. All patients not meeting these criteria are high-risk relapsing patients. *** For relapsed/refractory AML by risk groups, only 3-year OS was calculated as the high-risk group did not complete 5 years follow-up.

**Supplementary Table S11. Change in cost (in USD and EGP) per change in survival (ICER) between patients diagnosed in 2013 and 2017**

| **Cancer type** | **3-year Costs in EGP (USD) (2013–2017)** | | | | **3-year Overall survival (OS) (%)** | | | | **ICER * (USD)**  **Δ Cost**  **Δ OS** | **ICER * (EGP)**  **Δ Cost**  **Δ OS** |
| --- | --- | --- | --- | --- | --- | --- | --- | --- | --- | --- |
|  | **Costs in 2013**  **EGP (USD)** | **Costs in 2017**  **EGP (USD)** | **Percent of change in cost**  **USD% (EGP%)** | ***p*-value ^a^** | **OS (2013)** | **OS (2017)** | **Diff. OS** | ***p*-value ^b^** |  |  |
| All cancers combined | 316,228 ($22,117) | 395,302 ($16,409) | -25.8% (+25.0%) | <0.001* | 74.1 | 78.7 | 4.6 | <0.001* | (1,241) | 17,190 |
| 1. Leukaemia | 561,615 ($37,953) | 642,547 ($26,822) | -29.3% (+14.4%) | <0.001* | 74.5 | 82.8 | 8.3 | <0.001* | (1,341) | 9,751 |
| Ia. Acute Lymphoid Leukaemia | 526,062 ($35,291) | 621,270 ($25,938) | -26.5% (+18.1%) | <0.001* | 80.8 | 87.7 | 6.9 | 0.007* | (1,356) | 13,798 |
| Ib. Acute Myeloid Leukaemia | 727,116 ($52,052) | 768,337 ($31,559) | -39.4% (+5.7%) | <0.001* | 56.6 | 67.9 | 11.3 | 0.090 | (1,814) | 3,648 |
| Ic. Chronic Myeloid Leukaemia | 659,469 ($46,579) | 400,420 ($17,078) | -63.3% (-39.3%) | 0.8 | 76.9 | 93.3 | 16.4 | 0.210 | (1,799) | -15,796 |
| Id. Myelodysplastic Syndrome | 715,699 ($50,136) | 777,906 ($32,330) | -35.5% (+8.7%) | 0.003* | 50.1 | 20.1 | -30.0 | 0.672 | 594 | -2,074 |
| Ie. Juvenile myelomonocytic Leukaemia | 337,140 ($21,488) | 481,838 ($20,456) | -4.8% (+42.9%) | <0.001* | 58.3 | 53.8 | - 4.5 | 0.721 | 229 | -32,155 |
| 1. Lymphomas | 169,760 ($12,222) | 174,237 ($7,029) | -42.5% (+2.6%) | <0.001* | 86.9 | 94.8 | 7.9 | <0.001* | (657) | 567 |
| IIa. Hodgkin Lymphoma | 89,629 ($6,035) | 117,920 ($4,914) | -18.6% (+31.6%) | <0.001* | 97.2 | 98.2 | 1.0 | 0.330 | (1,121) | 28,291 |
| IIb. Non-Hodgkin Lymphoma | 356,426 ($24,564) | 405,375 ($16,661) | -32.2% (+13.7%) | <0.001* | 77.6 | 90.3 | 12.7 | 0.004* | (622) | 3,854 |
| III. Brain Tumours | 217,627 ($15,333) | 229,396 ($9,596) | -37.4% (+5.4%) | <0.001* | 64.2 | 69.9 | 5.7 | 0.12 | (1,007) | 2,065 |
| IV. Other Solid Tumours | 243,722 ($17,206) | 349,040 ($14,514) | -15.6% (+43.2%) | <0.001* | 72.3 | 73.1 | 0.8 | 0.65 | (3,365) | 131,648 |
| IV.a Neuroblastoma | 411,318 ($27,855) | 548,786 ($23,079) | -17.1% (+33.4%) | <0.001* | 66.7 | 55.4 | -11.3 | 0.045* | 423 | -12,165 |
| V. Retinoblastoma | 103,388 ($6,873) | 130,657 ($5,498) | -20.0% (+26.4%) | <0.001* | 91.6 | 97.3 | 5.7 | 0.058 | (241) | 4,784 |
| VI. Renal Tumours | 145,679 ($9,911) | 233,742 ($9,779) | -1.3% (+60.5%) | <0.001* | 89.7 | 85.9 | - 3.8 | 0.510 | 35 | -23,175 |
| VII.a Hepatoblastoma** | 186,139 ($13,020) | 359,388 ($15,145) | 16.3% (+93.1%) | <0.001* | 52.8 | 80.2 | 27.4 | 0.018* | 78 | 6,323 |
| VIIIa Osteosarcoma | 528,464 ($37,198) | 534,598 ($22,256) | -40.2% (+1.2%) | <0.001* | 45.2 | 43.1 | - 2.1 | 0.490 | 7,115 | -2,921 |
| VIIIc Ewing sarcomas | 403,408 ($27,379) | 567,453 ($23,231) | -13.0% (+40.7%) | <0.001* | 58.1 | 77.6 | 19.5 | 0.012* | (182) | 8,413 |
| IXa. Rhabdomyosarcoma | 219,125 ($14,963) | 340,411 ($13,969) | -6.6% (+55.4%) | <0.001* | 68.1 | 63.8 | - 4.3 | 0.810 | 231 | -28,206 |
| IXb-d Other soft tissue tumours | 334,523 ($23,792) | 236,667 ($9,681) | -59.3% (-29.3%) | 0.5 | 87.5 | 74.1 | -13.4 | 0.291 | 1,053 | 7,303 |
| X. a-c Germ Cell Tumours | 216,988 ($14,736) | 202,156 ($8,574) | -41.8% (-6.8%) | 0.1 | 81.5 | 89.5 | 8.0 | 0.401 | (770) | 1,854 |
| XII. Other Tumours (LCH)*** | 114,713 ($7,970) | 146,934 ($5,894) | -26.0% (+28.1%) | <0.001* | 85 | 88.4 | 3.4 | 0.690 | (610) | 9,477 |

*****ICER was calculated as incremental costs (difference between median costs of patients diagnosed in 2017 compared with those diagnosed in 2013) divided by the incremental survival (difference between the 3-year overall survival of patients diagnosed in 2017 compared with those diagnosed in 2013).

^a^ P-value for difference in costs was obtained from Wilcoxon**.** ^b^ *P-*value for difference in survival was obtained from log-rank test.

**Supplementary Table S12. Disease-related characteristics for patients diagnosed in 2013 versus 2017**

| **Cancer type** | **Cancer stage/risk/subtype at initial diagnosis** | **Patients diagnosed in 2013** | | **Patients diagnosed in 2017** | | **Diff. in Percent**  **(2017 – 2013)** |
| --- | --- | --- | --- | --- | --- | --- |
|  |  | **No.** | **Percent** | **No.** | **Percent** |  |
| 1. **Leukaemia** | | | | |  |  |
| Ia. Acute Lymphoid Leukaemia – Risk | Low risk | 128/276 | 46.3% | 203/460 | 44.1% | -2.20% |
|  | Standard risk | 121/276 | 43.8% | 226/460 | 49.1% | 5.30% |
|  | High risk | 27/276 | 9.7% | 31/460 | 6.7% | -3.00% |
| Ib. Acute Myeloid Leukaemia – Risk | Low | 28/81 | 34.5% | 39/128 | 30.4% | -4.10% |
|  | Intermediate | 37/81 | 45.6% | 70/128 | 54.7% | 9.10% |
|  | High | 16/81 | 19.7% | 19/128 | 14.8% | -4.90% |
| Ic. Chronic Myeloid Leukaemia – Subtype | Chronic phase | 13/13 | 100% | 14/15 | 93.3% | -6.70% |
|  | Blast crisis | 0/13 | 0% | 1/15 | 6.6% | 6.60% |
|  | Accelerated | 0/13 | 0% | 0/15 | 0% | 0.00% |
| Id. Myelodysplastic Syndrome – Risk | Low | 1/8 | 12.5% | 1/6 | 16.6% | 4.10% |
|  | Intermediate | 2/8 | 25% | 1/6 | 16.6% | -8.40% |
|  | High | 3/8 | 37.5% | 2/6 | 33.3% | -4.20% |
|  | Very high | 2/8 | 25% | 2/6 | 33.3% | 8.30% |
| 1. **Lymphomas** | | | | |  |  |
| IIa. Hodgkin Lymphoma – Risk | Low | 53/109 | 48.6% | 93/175 | 53.1% | 4.50% |
|  | Intermediate | 20/109 | 18.3% | 34/175 | 19.4% | 1.10% |
|  | High | 36/109 | 33.0% | 48/175 | 27.4% | -5.60% |
| IIb. Non-Hodgkin Lymphoma – Stage | Stage I | 6/121 | 4.9% | 6/136 | 4.4% | -0.50% |
|  | Stage II | 17/121 | 14.0% | 17/136 | 12.5% | -1.50% |
|  | Stage III | 61/121 | 50.4% | 74/136 | 54.4% | 4.00% |
|  | Stage IV | 37/121 | 30.6% | 39/136 | 28.7% | -1.90% |
| 1. **CNS/Brain Tumours** – Subtype | Astrocytoma | 66/233 | 28.3% | 99/397 | 24.9% | -3.40% |
|  | Ependymoma | 24/233 | 10.3% | 45/397 | 11.3% | 1.00% |
|  | Medulloblastoma/Embryonal | 50/233 | 21.4% | 82/397 | 20.6% | -0.80% |
|  | Neuronal & Mixed Neuronal | 15/233 | 6.4% | 23/397 | 5.8% | -0.60% |
|  | Optic Gliomas | 3/233 | 1.3% | 23/397 | 5.8% | 4.50% |
|  | Brain stem lesions | 42/233 | 18.0% | 58/397 | 14.6% | -3.40% |
|  | Others** | 33/233 | 14.1% | 67/397 | 16.8% | 2.70% |
| **Other Solid Tumours** | | | | | |  |
| IV.a Neuroblastoma – Risk | Low | 3/161 | 1.8% | 14/217 | 6.4% | 4.60% |
|  | Intermediate | 60/161 | 37.2% | 54/217 | 24.8% | -12.40% |
|  | High | 98/161 | 60.9% | 149/217 | 68.6% | 7.70% |
| Neuroblastoma – Stage | Stage 1 | 3/161 | 1.8% | 6/217 | 2.7% | 0.90% |
|  | Stage 2 | 0/161 | 0% | 8/217 | 3.6% | 3.60% |
|  | Stage 3 | 60/161 | 37.3% | 63/217 | 29.0% | -8.30% |
|  | Stage 4 | 89/161 | 55.3% | 129/217 | 59.4% | 4.10% |
|  | Stage 4s | 9/161 | 5.6% | 11/217 | 5.1% | -0.50% |
| V. Retinoblastoma – Stage | Intra-ocular | 75/87 | 86.2% | 115/118 | 97.4% | 11.20% |
|  | Extra-ocular | 12/87 | 13.8% | 3/118 | 2.5% | -11.30% |
| VI. Renal Tumours – Subtype | Wilms tumours | 68/79 | 86.1% | 87/108 | 80.5% | -5.60% |
|  | Rhabdoid tumour | 1/79 | 1.3% | 3/108 | 2.7% | 1.40% |
|  | Clear Cell Sarcoma Kidney | 7/79 | 8.8% | 9/108 | 8.3% | -0.50% |
|  | Renal Cell Carcinoma | 1/79 | 1.3% | 3/108 | 2.7% | 1.40% |
|  | Others* | 2/79 | 2.5% | 6/108 | 5.5% | 3.00% |
| Renal Tumours – Stage | Stage I | 11/79 | 13.9% | 6/108 | 5.5% | -8.40% |
|  | Stage II | 10/79 | 12.6% | 20/108 | 18.5% | 5.90% |
|  | Stage III | 32/79 | 40.5% | 43/108 | 39.8% | -0.70% |
|  | Stage IV | 19/79 | 24.0% | 17/108 | 15.7% | -8.30% |
|  | Stage V | 7/79 | 8.9% | 9/108 | 8.3% | -0.60% |
| VII.a Hepatoblastoma – Stage | Stage I | 1/22 | 4.5% | 1/31 | 3.2% | -1.30% |
|  | Stage II | 0/22 | 0% | 1/31 | 3.2% | 3.20% |
|  | Stage III | 15/22 | 68.2% | 22/31 | 70.9% | 2.70% |
|  | Stage IV | 6/22 | 27.3% | 7/31 | 22.6% | -4.70% |
| VIIIa Osteosarcoma – Stage | Localized | 32/54 | 59.3% | 41/61 | 89.1% | 29.80% |
|  | Metastatic | 22/54 | 40.7% | 20/61 | 32.8% | -7.90% |
| VIIIc Ewing sarcomas – Stage | Localized | 26/44 | 59.1% | 56/72 | 77.7% | 18.60% |
|  | Metastatic | 18/44 | 40.9% | 16/72 | 22.2% | -18.70% |
| IXa. Rhabdomyosarcoma – Stage | Stage I | 5/49 | 10.2% | 19/70 | 27.1% | 16.90% |
|  | Stage II | 6/49 | 12.2% | 2/70 | 2.8% | -9.40% |
|  | Stage III | 22/49 | 44.9% | 33/70 | 47.1% | 2.20% |
|  | Stage IV | 16/49 | 32.6% | 14/70 | 20.0% | -12.60% |
| IXb-d Other soft tissue tumours – Risk | Low | 2/8 | 25% | 7/24 | 29.2% | 4.20% |
|  | Intermediate | 4/8 | 50% | 14/24 | 58.3% | 8.30% |
|  | High | 2/8 | 25% | 3/24 | 12.5% | -12.50% |
| X. a-c Germ Cell Tumours – Risk | Low | 2/22 | 9.1% | 16/43 | 37.2% | 28.10% |
|  | Intermediate | 2/22 | 9.1% | 6/43 | 13.9% | 4.80% |
|  | High | 16/22 | 72.7% | 21/43 | 48.8% | -23.90% |
| X. a-c Germ Cell Tumours – Stage | Stage I | 5/22 | 22.7% | 16/43 | 37.2% | 14.50% |
|  | Stage II | 0/22 | 0% | 5/43 | 11.6% | 11.60% |
|  | Stage III | 14/22 | 63.6% | 15/43 | 34.8% | -28.80% |
|  | Stage IV | 3/22 | 13.6% | 7/43 | 16.3% | 2.70% |
| XII. LCH*** | Multisystem RO- LR | 4/20 | 20.0% | 15/45 | 33.3% | 13.30% |
|  | Multisystem RO+ HR | 4/20 | 20.0% | 6/45 | 13.3% | -6.70% |
|  | Uni-system Multifocal | 5/20 | 25.0% | 9/45 | 20.0% | -5.00% |
|  | Uni-system Unifocal | 7/20 | 35.0% | 15/45 | 33.3% | -1.70% |

* Other CNS/brain tumours include: Other specified and unspecified intracranial and intraspinal neoplasms. NA refers to ‘not available’, defined as patients with undetermined risk at diagnosis, as these patients died before risk determination, as follows: AML risk (n=27, 4.9%). ‘X’ refers to the sub-groups of patients who did not complete 5 years of follow-up, or had no deaths or survivors within this group, and for which 5-year survival could not be calculated. Abbreviations: APL: Acute Promyelocytic Leukaemia; BMT: Bone Marrow Transplant; RAEB: Refractory anaemia with excess blasts; RC: refractory cytopenia. a–y refer to references for definitions of staging, risk stratification and sub-type classifications systems are listed in Supplementary Table S4.

**Supplementary Table S13. Hazard ratio (95% CI) estimated in Cox regression model for the association between costs and risk of mortality controlling for confounders, for all cancers combined and four main cancer types (who survived >1 year)**

| **Variable** | **HR (95% CI)** | **Coefficient** | ***p*-value** |
| --- | --- | --- | --- |
| **All cancers combined (n=7,667)** | |  |  |
| **Costs of treatment** (1,000 USD)** | 1.025 (1. 023 – 1.028) | 0.025 | <0.001* |
| **Sex** |  |  |  |
| Female | Ref. group | Ref. group | Ref. group |
| Male | 0.931 (0.832 – 1.043) | -0.071 | 0.219 |
| **Year of diagnosis** | 0.947 (0.908 – 1.989) | -0.053 | 0.014* |
|  | | | |
| **Leukaemia (n= 2,067)** | | | |
| **Costs of treatment** (1,000 USD)** | 1.012 (1.006 – 1.02) | 0.011 | <0.001* |
| **Sex** |  |  |  |
| Female | Ref. group | Ref. group | Ref. group |
| Male | 0.862 (0.684 – 1.09) | -0.148 | 0.213 |
| **Year of diagnosis** | 0.937 (0.859 – 1.02) | -0.065 | 0.142 |
| **Age at diagnosis** |  |  |  |
| 0-4 years | Ref. group | Ref. group | Ref. group |
| 5-9 years | 0.839 (0.636 – 1.11) | -0.175 | 0.221 |
| 10-14 years | 0.849 (0.608 – 1.19) | -0.164 | 0.344 |
| 15-18 years | 1.161 (0.758 – 1.78) | 0.148 | 0.493 |
| **Relapse/refractory status** |  |  |  |
| No | Ref. group | Ref. group | Ref. group |
| Yes | 20.865 (16.287 – 26.73) | 3.038 | <0.001* |
|  |  |  |  |
| **Lymphomas (n= 1,344)** | | | |
| **Costs of treatment** (1,000 USD)** | 1.035 (1.024 – 1.045) | 0.033 | <0.001* |
| **Sex** |  |  |  |
| Female | Ref. group | Ref. group | Ref. group |
| Male | 0.565 (0.336 – 0.958) | -0.571 | 0.033* |
| **Year of diagnosis** | 0.810 (0.660 – 0.994) | -0.211 | 0.044* |
| **Age at diagnosis** |  |  |  |
| 0-4 years | Ref. group | Ref. group | Ref. group |
| 5-9 years | 1.088 (0.526 – 2.248) | 0.084 | 0.717 |
| 10-14 years | 0.898 (0.430 – 1.873) | -0.107 | 0.856 |
| 15-18 years | 1.138 (0.473 – 2.741) | 0.129 | 0.588 |
| **Relapse/refractory status** |  |  |  |
| No | Ref. group | Ref. group | Ref. group |
| Yes | 12.928 (7.718 – 21.654) | 2.559 | <0.001* |
|  |  |  |  |
| **Brain Tumours (n= 1,659)** | | | |
| **Costs of treatment** (1,000 USD)** | 1.017 (1.003 – 1.03) | 0.016 | 0.019* |
| **Sex** |  |  |  |
| Female | Ref. group | Ref. group | Ref. group |
| Male | 0.912 (0.714 – 1.16) | -0.092 | 0.459 |
| **Year of diagnosis** | 1.029 (0.934 – 1.13) | 0.028 | 0.566 |
| **Age at diagnosis** |  |  |  |
| 0-4 years | Ref. group | Ref. group | Ref. group |
| 5-9 years | 0.873 (0.659 – 1.16) | -0.135 | 0.345 |
| 10-14 years | 0.972 (0.698 – 1.35) | -0.028 | 0.866 |
| 15-18 years | 0.897 (0.512 – 1.57) | -0.108 | 0.704 |
| **Relapse/refractory status** |  |  |  |
| No | Ref. group | Ref. group | Ref. group |
| Yes | 7.079 (5.306 – 9.44) | 1.957 | <0.001* |
|  |  |  |  |
| **Other Solid Tumours (n= 2,908)** | | | |
| **Costs of treatment** (1,000 USD)** | 1.026 (1.023- 1.03) | 0.025 | <0.001* |
| **Sex** |  |  |  |
| Female | Ref. group | Ref. group | Ref. group |
| Male | 0.917 (0.780 – 1.080) | -0.085 | 0.301 |
| **Year of diagnosis** | 0.956 (0.898 – 1.021) | -0.044 | 0.170 |

* Significant at *p*-value <0.05. In Cox PH models for all cancers combined and for the solid tumours group, the ‘age at diagnosis’ and ‘diagnostic group’ variables were stratified in the Cox model using the *strata*() argument for which there are no hazards ratios for the stratification variables.
