# Supplementary material for: Cost-effectiveness of childhood cancer treatment in Egypt: Lessons to promote high-value care in a resource-limited setting based on real-world evidence
Source: eClinicalMedicine. 2022 Nov 4;55:101729. doi: 10.1016/j.eclinm.2022.101729 (PMC9646894; doi:10.1016/j.eclinm.2022.101729)
Supplement: Caption for Supplementary Materials [file mmc2.docx]

**Caption for supplementary materials**

**S1. Supplementary Methods**

S1.1. Coding of childhood cancer diagnoses

S1.2. Adopted treatment protocols for children with cancer

S1.3. Costing approach

S1.4. Disability-adjusted life years (DALY) calculation

S1.5. Sensitivity analysis

S1.6. ICER calculation

**Supplementary Table S1. Childhood cancer types grouped by International Classification of Childhood Cancers, third edition (ICCC-3) diagnostic groups**

**Supplementary Table S2. Standard Treatment protocols adopted at CCHE**

**Supplementary Table S3. Definitions of cost categories/sub-categories, and methods of measurement and/or allocation**

**Supplementary Table S4. Definitions of stage at diagnosis, risk stratification and sub-type classifications with references.**

**Supplementary Table S5. The Consolidated Health Economic Evaluation Reporting Standards (CHEERS) 2022 checklist**

**Supplementary Table S6. Base-case and sensitivity analysis for Cost per DALY averted, (N=8,886)**

**Supplementary Table S7. Childhood cancer costs at 3-years post-diagnosis (2013–2017), by major cost categories (N=8,886)**

**Supplementary Table S8. Cost per DALY averted for childhood cancers, stratified by stage, risk, or subtype (N=8,886)**

**Supplementary Table S9. Cost per DALY averted for childhood cancers, stratified by relapse/refractory (R/R) or progressive disease (PD) status**

**Supplementary Table S10. Cost per DALY averted for relapsed acute leukaemia (ALL/AML), by disease characteristics and BMT status**

**Supplementary Table S11. Change in cost (in USD and EGP) per change in survival (ICER) between patients diagnosed in 2013 and 2017**

**Supplementary Table S12. Disease-related characteristics for patients diagnosed in 2013 versus 2017**

**Supplementary Table S13. Hazard ratio (95% CI) estimated in Cox regression model for the association between costs and risk of mortality controlling for confounders, for all cancers combined and four main cancer types (who survived >1 year)**
